# Supplementary material for: High PrEP uptake and objective longitudinal adherence among HIV-exposed women with personal or partner plans for pregnancy in rural Uganda: A cohort study
Source: PLoS Med. 2023 Feb 16;20(2):e1004088. doi: 10.1371/journal.pmed.1004088 (PMC9983833; doi:10.1371/journal.pmed.1004088)
Supplement: S1 Text — (DOCX) [file pmed.1004088.s001.docx]

**S1 Text**

**Supporting Information 1:** Table 1 Scoring Details

**Parenthood Motivation**

A score was derived by summing over 3 statements related to each subscale as follows:

- ***Happiness***: (1) “It is nice to have children around” + (2) “I want to have a unique relationship with the child”’ + (3) “Bring up children brings happiness”
- ***Well-being***: (1) “Parenthood makes the relationship with your partner complete” + (2) “Children make life complete” + (3) “Parenthood gives you a goal to live for
- ***Identity***: (1) “It is obvious to have children” + (2) “Parenthood is a sign of being grown up” + (3) “Parenthood is the nature of women”
- ***Parenthood***: (1) “Parenthood fulfills motherly feelings” + (2) “Parenthood is satisfying” + (3) “I want to experience pregnancy and birth”
- ***Social Control***: (1) “My environment (others, family) expects it of me” + (2) “Others around me have children” + (3) “I want to have a baby to avoid being an outsider”
- ***Continuity***: (1) “Parenthood allows a person to continue the family name/tradition” + (2) “Parenthood allows a person not to be alone when you are old” + (3) “I want to have something of myself that continues living after I die”

**Reproductive Autonomy**

- ***Free from Coercion*** responses to the following 5 statements were scored as (1) for ‘strongly agree’, (2) for ‘agree’, (3) for ‘disagree’, (4) for ‘strongly disagree’: (1) “My pregnancy partner has stopped me from using a method to prevent pregnancy when I wanted to use one”, (2) “My pregnancy partner has messed with or made it difficult to use a method to prevent pregnancy.”, (3) “My pregnancy partner has made me use a method to prevent pregnancy when I did not want to use one.”, (4) “If I wanted to use a method to prevent pregnancy, my desired pregnancy partner would stop me.”, and (5) “My pregnancy partner has pressured me to become pregnant.”
- ***Communication*** responses to the following 5 statements were scored as (1) for ‘strongly disagree’, (2) for ‘disagree’, (3) for ‘agree’, (4) for ‘strongly agree’: (1) “My pregnancy partner would support me if I wanted to use a method to prevent pregnancy.”, (2) “It is easy to talk about sex with my pregnancy partner.”, (3) “If I didn’t want to have sex with I could tell my pregnancy partner.”, (4) “If I was worried about being pregnant or not being pregnant, I could talk to my pregnancy partner about it.”, (5) “If I really did not want to become pregnant, I could get my pregnancy partner to agree with me.”
- ***Decision making*** responses to the following 4 statements were scores as (1) for ‘My partner or someone else’, (2) for ‘Me and my pregnancy partner (or someone else) equally’, (3) for ‘Me’: (1) “Who has the most say about whether you use a method to prevent pregnancy?”, (2) “Who has the most say about which method you would use to prevent pregnancy?”, (3) “Who as the most say about when you have a baby in your life?”, and (4) “If you become pregnant but it was unplanned, who would have the most say about whether you would raise the child, seek adoptive parents, or have an abortion?”

**Perceived HIV Risk**

- (1) “What is your gut feeling about how likely you are to get infected with HIV?” *[responses range from (1) Extremely unlikely to (4) Extremely likely]*
- (2) “I worry about getting infected with HIV” *[responses range from (1) Never to (4) All the time]*
- (3) “Getting HIV is something I am….” *[responses range from ‘Not concerned about’ to ‘Extremely concerned about’]*
- (4) “I am sure I will not get infected with HIV.” *[responses range from (1) ‘Strongly agree’ to (4) ‘Strongly disagree’]*
- (5) “I feel I am unlikely to get infected with HIV.” *[responses range from (0) Strongly agree through (5) Strongly disagree]*

(6) “I feel vulnerable to HIV infection.” *[responses range from (5) Strongly agree through (0) Strongly disagree]*
