# Supplementary material for: High PrEP uptake and objective longitudinal adherence among HIV-exposed women with personal or partner plans for pregnancy in rural Uganda: A cohort study
Source: PLoS Med. 2023 Feb 16;20(2):e1004088. doi: 10.1371/journal.pmed.1004088 (PMC9983833; doi:10.1371/journal.pmed.1004088)
Supplement: S2 Text — (DOCX) [file pmed.1004088.s002.docx]

**S2 Text**

**Supporting Information 2:** Full Models for Table 2

| **Table A. Adherence through 91 days measured through Wisepill: Age** | | | |
| --- | --- | --- | --- |
|  | **Adjusted models** | | |
| **Covariate** | **n** | **Est (95% CI)** | **p-value** |
| Age per 5 years | 101 | 1.69% (-1.71%, 5.09%) | 0.33 |

| **Table B. Adherence through 91 days measured through Wisepill: Education** | | | |
| --- | --- | --- | --- |
|  | **Adjusted models** | | |
| **Covariate** | **n** | **Est (95% CI)** | **p-value** |
| Some secondary education or higher (vs none or primary education) | 101 | 2.10% (-4.80%, 9.01%) | 0.55 |
| Depression score (>1.75 vs <= 1.75) | 101 | -5.47% (-12.60%, 1.65%) | 0.13 |

| **Table C. Adherence through 91 days measured through Wisepill: Number of live births** | | | |
| --- | --- | --- | --- |
|  | **Adjusted models** | | |
| **Covariate** | **n** | **Est (95% CI)** | **p-value** |
| Number of live births (2+ vs 0,1) | 101 | 1.00% (-6.86%, 8.85%) | 0.80 |
| Age per 5 years | 101 | 1.86% (-1.87%, 5.59%) | 0.33 |
| Depression score (>1.75 vs <= 1.75) | 101 | -5.75% (-12.84%, 1.34%) | 0.11 |
| Some secondary education or higher (vs none or primary education) | 101 | 2.11% (-4.82%, 9.05%) | 0.55 |
| Reproductive Autonomy subscale: Decision-making | 101 | -3.50% (-10.37%, 3.37%) | 0.32 |

| **Table D. Adherence through 91 days measured through Wisepill: Depression** | | | |
| --- | --- | --- | --- |
|  | **Adjusted models** | | |
| **Covariate** | **n** | **Est (95% CI)** | **p-value** |
| Depression score (>1.75 vs <= 1.75) | 101 | -5.60% (-12.72%, 1.53%) | 0.12 |

| **Table E. Adherence through 91 days measured through Wisepill: Parenthood motivation subscale: Social Control** | | | |
| --- | --- | --- | --- |
|  | **Adjusted models** | | |
| **Covariate** | **n** | **Est (95% CI)** | **p-value** |
| Parenthood motivation subscale: Social Control | 101 | 0.04% (-1.87%, 1.95%) | 0.97 |
| Depression score (>1.75 vs <= 1.75) | 101 | -5.62% (-12.90%, 1.67%) | 0.13 |
| Age per 5 years | 101 | 1.83% (-1.96%, 5.63%) | 0.34 |
| Reproductive Autonomy subscale: Decision-making | 101 | -3.58% (-10.67%, 3.50%) | 0.32 |
| Number of live births (2+ vs 0,1) | 101 | 1.10% (-6.96%, 9.15%) | 0.79 |
| Some secondary education or higher (vs none or primary education) | 101 | 2.08% (-4.87%, 9.03%) | 0.56 |
| Sexual Relationship Power Scale | 101 | 0.45% (-5.06%, 5.95%) | 0.87 |

| **Table F. Adherence through 91 days measured through Wisepill: Sexual Relationship Power Scale** | | | |
| --- | --- | --- | --- |
|  | **Adjusted models** | | |
| **Covariate** | **n** | **Est (95% CI)** | **p-value** |
| Sexual Relationship Power Scale | 101 | 0.38% (-5.10%, 5.86%) | 0.89 |
| Depression score (>1.75 vs <= 1.75) | 101 | -5.73% (-12.93%, 1.46%) | 0.12 |
| Reproductive Autonomy subscale: Decision-making | 101 | -3.63% (-10.62%, 3.36%) | 0.31 |
| Age per 5 years | 101 | 2.03% (-1.37%, 5.44%) | 0.24 |
| Some secondary education or higher (vs none or primary education) | 101 | 1.93% (-4.94%, 8.80%) | 0.58 |

| **Table G. Adherence through 91 days measured through Wisepill: Reproductive Autonomy subscale: Decision-making** | | | |
| --- | --- | --- | --- |
|  | **Adjusted models** | | |
| **Covariate** | **n** | **Est (95% CI)** | **p-value** |
| Reproductive Autonomy subscale: Decision-making | 101 | -3.23% (-10.19%, 3.73%) | 0.36 |
| Age per 5 years | 101 | 1.95% (-1.48%, 5.38%) | 0.26 |

| **Table H. Adherence through 91 days measured through Wisepill: Perceived HIV Risk Score** | | | |
| --- | --- | --- | --- |
|  | **Adjusted models** | | |
| **Covariate** | **n** | **Est (95% CI)** | **p-value** |
| Perceived HIV Risk Score | 101 | 0.04% (-1.22%, 1.30%) | 0.95 |
| Age per 5 years | 101 | 1.88% (-1.89%, 5.66%) | 0.33 |
| Depression score (>1.75 vs <= 1.75) | 101 | -5.77% (-12.87%, 1.34%) | 0.11 |
| Reproductive Autonomy subscale: Decision-making | 101 | -3.48% (-10.37%, 3.41%) | 0.32 |
| Some secondary education or higher (vs none or primary education) | 101 | 2.13% (-4.83%, 9.10%) | 0.55 |
| Number of live births (2+ vs 0,1) | 101 | 0.96% (-6.96%, 8.89%) | 0.81 |
