# Supplementary material for: High PrEP uptake and objective longitudinal adherence among HIV-exposed women with personal or partner plans for pregnancy in rural Uganda: A cohort study
Source: PLoS Med. 2023 Feb 16;20(2):e1004088. doi: 10.1371/journal.pmed.1004088 (PMC9983833; doi:10.1371/journal.pmed.1004088)
Supplement: S3 Text — (DOCX) [file pmed.1004088.s003.docx]

**S3 Text**

**Supporting Information 3:** Summary of plasma, DBS, Wisepill data at 3-, 6- and 9-month visits

1. 3-month visit

| **Table A. 3-month data – all available** | | | | | | | |
| --- | --- | --- | --- | --- | --- | --- | --- |
|  | Wisepill (n=93) | | | Plasma (n=44) | | DBS (n=104) | |
| Level |  | 30 days prior 3 months | 60 days prior 3 months |  | 3 months |  | 3 months |
| Not detected | <1% | 2 (2%) | 1 (1%) | <=0.31 | 14 (32%) | <31.3 | 22 (21%) |
| Low | 1-<40% | 1 (1%) | 1 (1%) | >0.31-<10 | 0 | 31.3-<450 | 28 (27%) |
| Moderate | 40-80% | 11 (12%) | 8 (9%) | 10-40 | 1 (2%) | 450-<600 | 5 (5%) |
| High | >=80% | 79 (85%) | 83 (89%) | >=40 | 29 (66%) | >=600 | 49 (47%) |

| **Table B. 3-month data – restrict plasma and DBS to those that also have Wisepill data** | | | | |
| --- | --- | --- | --- | --- |
|  | Plasma (n=36) | | DBS (n=93) | |
| Level |  | 3 months |  | 3 months |
| Not detected | <=0.31 | 10 (28%) | <31.3 | 18 (19%) |
| Low | >0.31-<10 | 0 | 31.3-<450 | 24 (26%) |
| Moderate | 10-40 | 1 (3%) | 450-<600 | 5 (5%) |
| High | >=40 | 25 (70%) | >=600 | 46 (49%) |

Spearman correlations at 3 months:

- DBS and adherence 30-day prior is: 0.06 (p=0.55), n=93
- DBS and adherence 60-day prior is:  0.17 (p=0.11), n=93
- Plasma and adherence 30-day prior is: 0.44 (p=0.01), n=36

1. 6-month visit

| **Table C. 6-month data – all available** | | | | | | | |
| --- | --- | --- | --- | --- | --- | --- | --- |
|  | Wisepill (n=72) | | | Plasma (n=25) | | DBS (n=79) | |
| Level |  | 30 days prior 6 months | 60 days prior 3 months |  | 6 months |  | 6 months |
| Not detected | <1% | 3 (4%) | 2 (3%) | <=0.31 | 9 (36%) | <31.3 | 23 (29%) |
| Low | 1-<40% | 2 (3%) | 4 (6%) | >0.31-<10 | 0 | 31.3-<450 | 14 (18%) |
| Moderate | 40-80% | 10 (14%) | 10 (14%) | 10-40 | 2 (8%) | 450-<600 | 10 (13%) |
| High | >=80% | 57 (79%) | 56 (78%) | >=40 | 14 (56%) | >=600 | 32 (41%) |

| **Table D. 6-month data – restrict plasma and DBS to those that also have Wisepill data** | | | | |
| --- | --- | --- | --- | --- |
|  | Plasma (n=22) | | DBS (n=71) | |
| Level |  | 6 months |  | 6 months |
| Not detected | <=0.31 | 8 (36%) | <31.3 | 20 (28%) |
| Low | >0.31-<10 | 0 | 31.3-<450 | 11 (15%) |
| Moderate | 10-40 | 1 (5%) | 450-<600 | 9 (13%) |
| High | >=40 | 13 (59%) | >=600 | 31 (44%) |

Spearman correlations at 6 months:

- DBS and adherence 30-day prior is: 0.04 (p=0.71), n=71
- DBS and adherence 60-day prior is:  0.03 (p=0.82), n=71
- Plasma and adherence 30-day prior is: 0.43 (p=0.046), n=22

1. 9-month visit

| **Table E. 9-month data – all available** | | | | | | | |
| --- | --- | --- | --- | --- | --- | --- | --- |
|  | Wisepill (n=53) | | | Plasma (n=22) | | DBS (n=65) | |
| Level |  | 30 days prior 9 months | 60 days prior 9 months |  | 9 months |  | 9 months |
| Not detected | <1% | 0 | 0 | <=0.31 | 8 (36%) | <31.3 | 25 (38%) |
| Low | 1-<40% | 1 (2%) | 1 (2%) | >0.31-<10 | 0 | 31.3-<450 | 10 (15%) |
| Moderate | 40-80% | 6 (11%) | 8 (15%) | 10-40 | 4 (18%) | 450-<600 | 1 (2%) |
| High | >=80% | 46 (87%) | 44 (83%) | >=40 | 10 (45%) | >=600 | 29 (45%) |

| **Table F. 9-month data – restrict plasma and DBS to those that also have Wisepill data** | | | | |
| --- | --- | --- | --- | --- |
|  | Plasma (n=17) | | DBS (n=65) | |
| Level |  | 9 months |  | 9 months |
| Not detected | <=0.31 | 6 (35%) | <31.3 | 19 (36%) |
| Low | >0.31-<10 | 0 | 31.3-<450 | 7 (13%) |
| Moderate | 10-40 | 3 (18%) | 450-<600 | 1 (2%) |
| High | >=40 | 8 (47%) | >=600 | 26 (49%) |

Spearman correlations at 9 months:

- DBS and adherence 30-day prior is: -0.10 (p=0.48), n=53
- DBS and adherence 60-day prior is:  -0.04 (p=0.76), n=53
- Plasma and adherence 30-day prior is: 0.49 (p=0.045), n=17
