# Supplementary material for: High PrEP uptake and objective longitudinal adherence among HIV-exposed women with personal or partner plans for pregnancy in rural Uganda: A cohort study
Source: PLoS Med. 2023 Feb 16;20(2):e1004088. doi: 10.1371/journal.pmed.1004088 (PMC9983833; doi:10.1371/journal.pmed.1004088)
Supplement: S1 Protocol — (PDF) [file pmed.1004088.s005.pdf]

Principal Investigator: Lynn T. Matthews, MD MPH

Site PI: Mwebesa Bosco Bwana, MBChB MPH

Department/Division: MGH/ MUST

Protocol Title: Adherence to periconception HIV risk-reduction among uninfected women in rural Uganda

Protocol version date: 03 May 2018

I. BACKGROUND AND SIGNIFICANCE

Background

Uganda has one of the highest total fertility rates in the world at 6.2 children per woman and HIV-prevalence among women attending antenatal care is ~ 11% [1, 2]. While services to prevent perinatal transmission are robust for HIV-infected pregnant woman, HIV prevention *prior to a desired pregnancy* is rarely addressed. Yet, at least 30-50% of men living with HIV in Uganda desire children [3-7] and nearly half have a stable, uninfected partner [8]. Women risk condomless sex in order to meet important personal and sociocultural goals to have children [3, 7, 9-11]. Integrating HIV prevention into reproductive health programs presents an opportunity to reduce HIV incidence among women in settings where fertility rates and HIV prevalence are high.

Several effective strategies are available to women who want to conceive with an at-risk partner who is engaged in care including delaying condomless sex until the infected partner is on effective ART, treating STIs in both partners, limiting condomless sex to peak fertility, and sperm processing [12-14]. However, in a setting where gender power imbalances make it challenging for a woman to insist that her partner participate in strategies to reduce sexual transmission of HIV, pre-exposure prophylaxis (PrEP) may be a critical safer conception strategy. Understanding how women initiate and adhere to PrEP as periconception risk reduction is an important step towards developing safer conception programs [15, 16].

Significance

Table 1. Safer conception methods for uninfected women with HIV-infected male partners.
Adapted from Matthews et al. *Curr Opinion HIV AIDS [12]*

Method	Est. risk reduction
Couples-based strategies	
ART for infected partner [17]	96%
STI treatment [18, 19]	≤ 40%
Sperm washing # [14, 20]	~100%
Condomless sex limited to peak fertility* [21]	Unknown
Couples-based HIV counseling and testing^ [22]	Unknown
Female-controlled strategy	
Oral TDF/FTC PrEP for uninfected partner [23-25]	63-75%

* Risk reduction effected by reduced exposure.
^ CHCT associated with decreased risk of HIV-acquisition. #Difficult to access.

1. <u>Reducing periconception HIV-acquisition risk is an efficient strategy to reduce HIV incidence among women and their children</u>. Integrating HIV prevention into reproductive health programs may reduce HIV incidence, particularly in settings like Uganda where fertility rates (6.2 children/woman) and HIV prevalence (7%) are high and HIV transmission within stable heterosexual relationships accounts for nearly half of new infections [26-28]. WHO and CDC guidelines emphasize that eliminating perinatal transmission requires pre-conception counseling to reduce transmission to the mother and therefore the child [29, 30]. Programs to incorporate safer conception counseling have not been developed.

2. <u>Safer conception tools are available</u>. Data suggest that men and women living with HIV, their uninfected partners, and HIV care providers are eager for implementation of safer conception programs in sub-Saharan Africa [7, 10, 11, 31]. Couples-based strategies such as ART for the infected partner, delaying

condomless sex until HIV RNA is suppressed, and sperm washing can reduce HIV transmission to negligible levels. Couples-based HIV-counseling and testing and STI treatment may further reduce transmission (Table 1). Pre-exposure prophylaxis (PrEP), which does not require partner participation, is an additional safer conception strategy, that reduces HIV-acquisition risk by as much as 94%.

3. PrEP gives uninfected women a strategy to protect herself (and therefore her child) from HIV. Women who partner with an HIV-infected man have limited power to negotiate safer sex or safer conception practices [3, 10, 32]. HIV-uninfected women partnered with men living with HIV describe obligations to meet childbearing expectations, irrespective of HIV risk [10]. In oral PrEP trials with high adherence, women experienced a reduction in HIV acquisition of 63-75% [23, 24]. Among women with plasma concentrations of tenofovir indicating recent dosing, protection was estimated at 94% [25]. While women who enrolled in PrEP trials without being in mutually-disclosed serodiscordant partnerships had particular challenges taking study drug [33, 34], studies since PrEP efficacy has been proven suggest that this population is able and willing to take daily, oral PrEP [35]. Many of the barriers to adherence described by VOICE and Fem-PrEP trial participants with low adherence related to lack of proven efficacy of study drug, low risk perceptions, and wanting study services without taking study drug [36, 37]. The current proposal avoids these pitfalls by communicating unequivocal PrEP efficacy to women who believe their partner is HIV-infected and plan to conceive a child. Furthermore, PrEP acceptance is not a requirement of the study, which minimizes incentives for initiating PrEP without intent to use PrEP.

Given the limited prevention options for women who choose to conceive with HIV-positive men, WHO guidelines highlight serodiscordant couples considering conception are a priority group for PrEP[38]. The FDA labeling information and the U.S. Perinatal ART Guidelines support periconception PrEP use [16, 30, 39-42]. However, there are no data on feasibility and uptake of this strategy in settings with generalized epidemics.

II. RESEARCH DESIGN AND SPECIFIC AIMS

Table 2. Conceptual framework for pregnancy-related PrEP adherence

Domain	Construct	Measure(s)
Individual	HIV Knowledge	HIV knowledge [43]
		Safer conception knowledge[44]
	Risk	Partner serostatus
		Risk perception [45]
		Risk behavior via diary
	Optimism	PrEP optimism [46]
	Motivation	Parenthood motivation [47]
	Medication side effects	
	Socio-demographics	e.g. asset index, substance use, reproductive history, partnership type
Couple	Partnership disclosure	HIV disclosure [48, 49]
		Complete couples-based HIV counseling and testing
	Communication, gender norms	Relationship power [50]
		Partner communication[51]

Community	Social support	Functional Support [52]
	Stigma	Attributed Stigma [53]

Research Design: In our mixed-methods research study we will offer comprehensive safer conception services to 150 HIV-uninfected women reporting an HIV-infected or high-risk partner and personal or partner plans for pregnancy in rural Uganda to evaluate prevalence and determinants of uptake and adherence (tenofovir plasma concentration $\geq$40ng/mL, opening pill device to take $\geq$80% of dispensed pills) to PrEP and other safer conceptions strategies among Ugandan women exposed to HIV.

To evaluate factors that we believe may be experienced by our target study population at an individual, couple, and community-level that may influence PrEP initiation and adherence behaviors we have created a conceptual framework (Table 2), adapted from van der Straten*et al.*[36, 54]. At the individual level, she may have excellent knowledge of safer conception strategies, be optimistic about PrEP efficacy, and perceive herself to be at risk for acquiring HIV. However, on the dyadic level, she and her partner do not communicate or trust one another, making it hard for her to adhere to a daily medication. If she feels that being HIV-infected is highly stigmatized in the community, she may be more motivated to participate in periconception PrEP. By collecting covariates that reflect constructs of this framework, our mixed methods data will allow us to develop this framework to inform an adherence intervention for women seeking periconception HIV-prevention.

Specific Aims: Based on our conceptual framework for periconception risk reduction and adherence to periconception PrEP[36, 54] we propose the following specific aims:

1. We will measure prevalence and factors associated with *uptake* of daily, oral TDF/FTC PrEP over 9 months. We hypothesize that 70% of women will initiate PrEP as part of safer conception and that uptake will be associated with greater relationship power and social support. Secondary outcomes include uptake of couples-based strategies including ART uptake by partners, limiting condomless sex to fertility, STI treatment, and couples-based HIV counseling and testing.
2. We will objectively measure *adherence* to PrEP over up to 9 months. We hypothesize that 70% of women will have detectable plasma drug 3 months after PrEP initiation and that 70% of women will have >80% adherence by electronic Wisepill monitoring. We hypothesize that women with greater relationship power and social support will be more likely to adhere. We will evaluate adherence to couples-based strategies including delay of condomless sex until partner taking ART for $\geq$3 months and limiting condomless sex to peak fertility. We will measure HIV and pregnancy incidence.
3. Based on a conceptual framework for PrEP adherence, we will conduct in-depth interviews with a subset of up to 45 women and their partners to explore barriers and promoters to use of PrEP and other safer conception strategies.
4. We will offer objective sexually transmitted infection (STI) testing to all participants in order to measure the prevalence, incidence, and factors associated with STIs. We will compare incidence among women who choose and who do not choose PrEP.

Offering women strategies to reduce HIV acquisition risk while meeting reproductive goals provides a unique approach to reducing HIV incidence among women of reproductive age and their children.

III. SUBJECT SELECTION

Self-Report Inclusion Criteria

1. Female
2. Aged 18-35
3. Likely to be fertile based on reproductive health history [55]
4. Reported personal or partner desire to have a child in the next year [56-59]
5. With a partner she reports as HIV-infected or likely to be HIV-infected (e.g. taking medicine daily, goes to clinic routinely, has HIV-infected partners, he has implied that he is “sick” but has not disclosed).
6. Live within 60km of clinic. Not planning on relocating to an area incompatible with ability to attend quarterly clinic over a 9-month follow-up period

Objective Inclusion Criteria

1. HIV-negative (onsite rapid testing)
2. Not currently pregnant (onsite urine b-hcg testing)
3. Fluent in English or local language
4. Otherwise able to participate in the informed consent process

Enrolment Criteria Male Partners

We will enroll a subset of male partners to participate in in-depth interviews. Inclusion criteria include:

1. Partnered with female participant
2. Fluent in English or local language
3. Otherwise able to participate in the informed consent process

Source of Subjects

We will recruit and identify potential participants who may meet our selection criteria from:

1. Our existing Healthy Families Program located at the Immune Suppression Syndrome (ISS) Clinic located in Mbarara town within the Mbarara District of Uganda. This clinical program currently recruits HIV-affected individuals and couples for safer conception counseling and service provision (exclusive of PrEP which is not currently available in the public sector).
2. MRRH HIV counseling and testing sites where > 300 women screen monthly and approximately 8% test HIV-positive
3. Community partnerships and referrals from local healthcare providers
4. Advertisement using flyers at our Safer Conception Clinic, local HIV counselling and testing sites, appropriate community events, and informational radio spots.
5. Male partners will be invited to participate by their female partners. Female participants who are interested in including their partners in the study will be given an invitation letter which will offer contact details should their male partner choose to contact the research team. He will also be invited to come to the clinic or research space alone or with his female partner.

Sample Size:

We will enroll up to 150 women who meet the outlined inclusion/exclusion criteria and complete an informed consent process. We will invite a subset (up to 45) of these women to participate in in-depth interviews. We will invite these women to invite their pregnancy partner for an in-depth interview (up to 45 partners.) We will enroll up to 150 women who meet the outlined inclusion/exclusion criteria to complete objective STI testing pending ethics approval for this amendment.

IV. SUBJECT ENROLLMENT

Informed Consent

Upon completion of the screening questions, women who remain eligible will be asked to participate in an informed consent process before enrollment in the study. Women who screen positive for HIV and/or pregnancy during the initial screening process will not be invited to participate in the informed consent process for this study and will be referred to antenatal and/or HIV care. They will still be eligible to participate in the clinical safer conception service.

Research Assistants that are certified in Good Clinical Practice will use a written informed consent document as a guide to provide potential participants information on study procedures and study expectations of the research participant. The informed consent will take place orally in a one on one interview held in a private space to allow potential participants the opportunity to ask questions of the study staff. The informed consent document will be available to the participant in both English and the local language. Translation of the informed consent (and all other participant relevant study documents) will be subjected to translation, back translation and revision to ensure accuracy, clarity and ease of comprehension. During the informed consent interview the research assistant will remind the potential participants that participation in the research study is completely optional and deciding not to participate will have no effect on the clinical services they receive.

The Informed Consent form will outline in detail:

- Explanation of the consenting process in compliance with institutional policies: statement that participation is voluntary
- Nature and purpose of study
- Length of the study and number of participants
- Explanation of procedures
- Discomforts and risks, plans to protect participants from these risks
- Benefits
- Alternatives to participating
- Rights as a participant of the study
- Financial arrangements and reimbursements
- Sources of additional information
- Confidentiality, including how data will be used and how it will be kept private
- Data storage and destruction.

After each major section and any key points, the Research Assistant will pause and check for understanding and provide further clarification if necessary. All participants will be informed that the information they provide through the consent, interviews, lab work and questionnaires will be

treated as confidential (i.e., not shared with anyone outside of the research staff with linkage to identifying information) and voluntary (i.e., they are not obligated to answer any question). If women prefer to take the screening and/or informed consent documents with them to further consider participation before completing informed consent for enrollment that will be presented as an option and study staff will check-in on their enrollment decision via the cell phone number provided by the women on their contact sheet.

V. STUDY PROCEDURES

Overview

Upon completing the informed consent process, interested participants will be asked to sign or fingerprint a copy of the written informed consent to confirm that they understand the study and what is expected of them as a research participant. A copy of the signed consent form will be given to the participant for their records. After signing of the consent, participants will be considered enrolled as part of the sample goal of 150 uninfected women.

Enrolled women will participate in the outlined mixed-method study to evaluate prevalence and determinants of uptake and adherence (tenofovir plasma concentration >40ng/mL, taking >80% of dispensed pills) to daily, oral TDF/FTC PrEP during periconception among HIV uninfected Ugandan women reporting HIV-infected or unknown serostatus partners. To accomplish this, women will participate in quarterly study visits, HIV and pregnancy testing, questionnaires and safer conception/adherence group counseling sessions. Participants will be eligible to initiate PrEP at any time during the first 6 months of the 9-month (maximum) study follow-up period. Participants will receive a PrEP referral form to bring to the clinic if they chose to initiate PrEP outside of a scheduled study follow up visit.

Women who become pregnant or test positive for HIV during the 9-month follow-up period will complete exit activities (questionnaire and in-depth interview) and be referred to appropriate antenatal and/or HIV follow-up care.

Counselling

Comprehensive safer conception counselling: Enrolled women will be offered a package of safer conception counselling for uninfected women who want to conceive a child with an infected partner:

- Encourage partner testing and serostatus disclosure within the partnership.
- Encourage partner to initiate ART if he is eligible by national guidelines (CD4 cell count ≤ 500 or in serodiscordant partnership),
- Delay sex without condoms until he achieves HIV viral load suppression or is on ART x 6 months,
- Consider contraception to delay pregnancy until safer conception strategies implemented
- Limit sex without condoms to peak fertility, and / or
- Consider sperm washing, donor sperm, and adoption as alternatives.

Safer conception counselling will occur at baseline and then quarterly thereafter in non-pregnant women

Adherence support counselling: Brief adherence counselling and support sessions will be available at to all women at the time of enrollment and will cover PrEP education. Follow-up adherence counselling will be available during quarterly visits to all women who initiate PrEP. Counselling will be performed by trained study counsellors in a individual counselling sesion. These sessions will discuss barriers to and promoters of adherence. **Data Collection (Table 3)**

Demographics: We will collect baseline demographic and reproductive health data (number of prior pregnancies, live births, living children) at enrollment. We will update demographic data at study exit to assess information that may change throughout the course of the study (e.g. employment, household income).

Measures: Constructs will be assessed with a questionnaire administered at 0 months and study exit (Table 2). *Other safer conception behaviors* will also be assessed, including partner HIV status, delay of sex without condoms until partner on ART with viral load suppression, accessing additional services such as sperm washing.

Sexual Behavior: Sexual behavior will be assessed through monthly diaries. The counsellor and the participant will fill in the first day of next anticipated menses on a monthly calendar and then participants will mark the number of condomless sex acts and bring this form back in for review at quarterly follow-up visits. We will use the sexual behavior data of woman who chose not to initiate PrEP to explore between group differences among individuals who chose to and who chose not to initiate PrEP. In addition, we will be able to better understand the entire sample's understanding and use of the alternative/additional safer conception strategy of timing sex to peak fertility by collecting this data regardless of PrEP initiation.

Table 3. Data collection procedures

Months	0	i*	3	6	9/stud exit
**Laboratory**					
Urine pregnancy test	x		x	x	x
HIV test	x		x	x	x
STI screen	x		x	x	x
STI Objective Testing	x			x	
STI Case Report Form	x			x	
Additional labs for women who initiate PrEP					
Creatinine		x	x	x	x
HBV serology		x			
Drug level adherence testing			x	x	x
**Behavioral**					
Safer conception counseling	x		x	x	x
Full questionnaire (See Table 2)	x				x
diary sexual behavior	x		x	x	x
Wisepill adherence data	x		x	x	x
In-depth interviews (See Aim 3)				x	x
Additional for women who initiate PrEP					
Adherence support	x	x	x	x	x

*i = PrEP initiation visit
** Objective STI Testing will include: gonorrhoeae, chlamydia, trichomonas vaginalis, herpes and syphilis at baseline and mont 6 for all participants and as needed based on screening at all other visits/exit

Biologic tests; All participants: All participants will complete quarterly urine pregnancy tests, rapid HIV tests and syndromic screening for STIs.

Beta hcg-urine pregnancy tests require approximately 4 drops of urine to test for human chorionic gonadotropin, a hormone that is produced once a woman becomes pregnant. Women who test positive for pregnancy after enrolment will be referred to appropriate antenatal care before being exited from the study.

Rapid HIV testing will occur via the standard of care finger-prick blood sample. HIV finger-prick testing will entail the clinical study member pricking the tip of the participant's

finger with a small, single-use lancet, squeezing a small amount of blood (approximately 1-2 drops) into a sample collection pipette, transferring a drop of blood into a marked sample well and waiting approximately 10-20 minutes for results. Women who test positive will receive a second, confirmatory finger-pick test. Upon completion of confirmatory testing, women who were confirmed positive will receive post-test counselling and be referred for appropriate HIV care before being exited from the study.

Syndromic STI screening: All women will be offered syndromic STI screening at each study visit. Syndromic STI screening will be conducted by asking participants if they have experienced any STI symptoms (e.g. discharge, ulcers, itching). For women who endorse STI symptoms a confirmatory test will be performed, and they will receive treatment medications dispensed by a licensed medical professional team member (see STI treatment below),

Objective STI testing: Women who consent to participate in objective STI testing will complete objective STI screening tests at baseline, at the 6-month study visit or study exit (if exit occurs prior to the 6-month timepoint) independent of the syndromic STI screen (see above).

Women who endorse symptoms of STI, as determined by syndromic STI screening, during interim visits or at the 3-month study visit will have additional objective STI testing.

Objective STI tests include testing for Chlamydia trachomatis, Neisseria gonorrhoeae, Trichomonas vaginalis, Herpes Simplex Virus 2 (HSV2), and Syphilis. At each testing timepoint women will provide 2 vaginal swab specimens by either self-collection or nurse collection (based on participant preference). If self-collection is selected, participants will receive detailed instruction from the nurse to ensure the sample will be viable for testing. Self-collection of vulvo-vaginal swabs among women in Sub-Saharan Africa with and without pregnancy has been found to be safe, reliable, acceptable, and efficient [19, 60].

Regardless of collection preference, all participants will be provided with a private environment for collection. Women will be asked to provide 2 plastic dacron swabs to facilitate objective testing for Chlamydia trachomatis, Neisseria gonorrhoeae, and Trichomonas vaginalis. The additional swab will be used to facilitate additional quality assurance testing as needed and stored for future testing by researchers involved with this study who, in the future, may think of other additional tests, related to our research questions pertaining to HIV risk, PrEP use and STIs, that they would like to perform on the additional swabs. Before we proceed with any future tests, we will submit the required proposals for ethics review by the university ethics review committee and the national council of science and technology.

Women will also provide a blood sample (1 tube, ~10cc or 1 tablespoon) to test for HSV2 Ab and Syphilis testing with Treponemal Ab and Venereal Disease Research Laboratory (non-trep) tests. We anticipate that up to 150 women (depending on start date) will undergo objective STI testing at the enrollment visit.

All biological tests including pregnancy testing, HIV testing, and STI syndromic screenings will be performed by clinical study staff in a private room located at the ISS clinic.

STI Treatment: Participants with positive STI testing will receive treatment at the study site dispensed by a licensed medical professional who is a member of the study team. All treatment regimens will be in accordance with standard Ugandan treatment guidelines. Study participants with positive STIs who receive treatment will also be given treatment medications to deliver to their partner(s) with the goal of preventing reinfection. This practice of patient-delivered partner medication has been shown to be efficacious in several settings, including Uganda [61].

Biologic tests; Women who initiate PrEP: Women who initiate PrEP will complete creatinine at baseline and quarterly and hepatitis B safety monitoring at baseline to ensure that there are no contraindications to using PrEP. A nurse will also assess for any symptoms experienced after initiating PrEP to evaluate for any medication side effects. Blood testing for renal function (Creatinine) and for hepatitis B infection will require 2 tubes of blood or approximately 10mL or <1 tablespoon of blood at baseline and 1 tube of blood or approximately 5mL quarterly. As per WHO guidelines, women will be eligible to initiate PrEP immediately. Women with abnormal renal function or active hepatitis B infection (HBV surface antigen positive) will be instructed to stop PrEP. Abnormal renal function will be defined as:

- Non pregnant women: serum Creatinine> 89 µmol/L and GFR < 60ml/min (Glomerular filtration will be estimated using the Cockcroft-Gault equation).

Any woman who has abnormal safety lab results will be referred to appropriate clinical care.

Adherence: Adherence of PrEP will be assessed by biological sampling as well as electronic data capture monitoring. In addition to assessing adherence we will promote adherence through offering individual adherence counselling sessions to all women who initiate PrEP at their study visits using an adherence counselling tool developed specifically for PrEP adherence.

Plasma tenofovir testing for plasma drug level will be collected at the first visit (3 months) after PrEP initiation and at exit or incident pregnancy. TFV concentration $\geq$40 ng/mL indicates adherence in the prior 24 hours and is associated with HIV protective efficacy (1° outcome) [25, 62]. We will collect additional samples to test for phosphorylated tenofovir (TFV-DP) via dried blood spots, which are whole blood and provide longer-term exposure information (half-life 17 days). DBS samples will be analyzed to adjudicate discrepancies between Wisepill and plasma TFV levels [63, 64].

In addition, a full blood count for hematocrit/hemoglobin testing will be taken at two instances:

- At the first instance of DBS collection (for TFV levels) after the participant has initiated PrEP for the first time,
- At the time of a positive pregnancy test if the participant is on PrEP and adherence DBS labs are indicated.

This hematocrit/hemoglobin testing is a point of care test and will not require additional blood sample to be taken. The hematocrit is required to accurately interpret the TFV level in the dried blood spot which is a primary adherence outcome for the study.

For participants who have already enrolled in the project we will test hematocrit/hemoglobin at their next study visit upon re-consenting them.

Wisepill device to monitor adherence. PrEP initiators will be given a Wisepill device to measure level and patterns of adherence in real-time. Wisepill uses wireless technology to monitor medication use. Device openings are recorded and transmitted via cellular networks in real-time to the Wisepill server. While not all device openings equate to ingestion, electronic adherence monitoring provides the best-available understanding of day-to-day adherence behavior. Flash memory maintains data for later transmission if connectivity is lost. We have collected Wisepill adherence data in Dr. Bangsberg's 750-person HIV cohort in Uganda (NCT01596322) and found a high degree of acceptability, reliability and validity with respect to predicting HIV RNA rebound [65]. Outcomes (median adherence >80%, median adherence, and number of interruptions >72 hours) will be calculated every 28 days and summarized quarterly.

Interviews: We will conduct qualitative interviews to explore factors affecting periconception PrEP uptake and adherence. We will sample from three groups of women (~15 per group): (1) women who do not initiate PrEP, (2) women who initiate PrEP and have detectable plasma TFV, and (3) women who initiate PrEP and have undetectable plasma TFV. Interviews will target adherence experiences of participants and cover a range of relevant topics, including: (a) circumstances under which pills are taken (dosing behavior); (b) missed doses and why they happen; (c) what obstacles impede adherence; (d) who/what helps with adherence and how; (e) how pregnancy plans influence adherence behavior, and (f) reasons for changes in adherence patterns over time with a focus on constructs within our conceptual framework. In addition, we will invite a subset of male pregnancy partners to participate in a similar in-depth interview to explore their experiences throughout the safer conception process.

VI. SAMPLE COLLECTION, STORAGE, USE AND SHIPMENT

Point of care testing: Urine pregnancy tests, rapid HIV tests and hematocrit testing will be performed at the MUST study clinic by study clinical staff. These tests are considered point-of-care tests and no samples will be stored or shipped. Sample specimens will be disposed of per the safety protocol of the study clinic in appropriately labelled biohazard waste receptacles.

Tenofovir testing

Number of samples: We are enrolling 150 women with personal or partner pregnancy desire to participate in the research project and offering the entire sample the option to initiate daily, oral PrEP. Based on enrolment and a maximum follow-up period of 9 months with quarterly adherence testing we estimate the maximum number of stored plasma samples to be 600. We anticipate collecting the DBS TNF-DP assay on the total number of samples for an estimated maximum of 600 DBS stored samples. The TNF-DP data will give a sense of longer-term adherence and help assess the "white coat" affect in this sample. Total temporarily-stored samples=1200.

Use: Whole blood will be drawn and prepared plasma samples will be stored to test tenofovir (TNF) concentration levels in plasma samples of women who initiated PrEP. In addition, dried blood spots (DBS) will be collected and stored to test tenofovir diphosphate (TNF-DP) concentration levels for all women who initiate PrEP. TNF-DP is a more sensitive assay and will allow for more granular data analysis and interpretation of plasma TNF concentration results.

Collection and sample preparation: Our collaborating lab at the Johns Hopkins Medical Institute requires a 4 ml K3EDTA tube of blood drawn at each time point for testing of plasma TNF. For plasma, the tube will be placed on ice after sampling and centrifuged down at 1500 g for ten minutes to separate the plasma. Immediate centrifugation negates the need for ice. Plasma will be stored in two aliquots at approximately -70°C within one hour of collection. Our collaborating lab at University of Colorado will perform DBS testing and requires, 50 uL of whole blood will be collected and spotted onto Whatman 903 protein saver cards using a calibrated research pipette. Spots will be dried completely at room temperature (2 hours out of direct sunlight) before sealing into a ziplock bag with a desiccant sachet and humidity indicator card. We will collect five spots (one card) per time point.

Shipment and Storage: All plasma and dried blood spot samples will be temporarily stored at approximately -70°C to ensure long-term stability. Shipping will occur in batches and be sent on dry ice to our collaborating lab at the Johns Hopkins Medical Institute in Baltimore, Maryland, United States or to University of Colorado, Denver, Colorado, United States. If extra samples are available, these materials may also be used by other researchers involved with this study who, in the future, may think of other additional tests, related to our research questions pertaining to HIV risk, PrEP use and STIs, that they would like to perform on the remaining samples within 10 years of collection. Before we proceed with any future tests, we will submit the required proposals for ethics review by the university ethics review committee and the national council of science and technology.

STI Testing (General):

All testing of vaginal swab samples for Gonorrhea, Chlamydia and Trichomonas will be processed at the MUST Microbiology lab via GeneXpert rapid nucleic acid amplification (NAAT) testing (see below.)

Testing of phlebotomy samples for Syphilis Treponemal Ab and non-treponemal as well as the preprocessing and serum extraction for HSV2 Ab testing will be performed at the MUST Research lab. (see below)

Serological testing for HSV2 (by ELISA) will be performed at Makerere University microbiology lab under Dr. Esther Joloba. Dr, Joloba's lab has expertise in performing ELISA serology testing for HSV2 and has published on the sensitivity and specificity of this assay methodology among HIV-infected and uninfected Ugandans [66].

Turnaround time for all testing (with the exception of HSV2 Ab) will be approximately two hours, allowing women with positive STI test results to be seen for follow-up clinical evaluation and treatment as well as counselling around partner referral and treatment at the same study visit. Women with an active outbreak of suspected HSV2 based on clinical exam will be offered acyclovir prior to objective confirmatory results as per standard of care.

All biological tests including pregnancy testing, HIV testing, and STI syndromic screenings will be performed by clinical study staff in a private room located at the ISS clinic.

STI Vaginal Swab Testing (MUST Microbiology Lab):

The MUST Microbiology lab has been fully functional for over ten years. The lab has expertise in various lab modalities including GeneXpert and run over 800 samples via GeneXpert each month which include TB sputum, TB CSF, TB stool, HIV RNA, and HPV testing ([67]). The MUST Microbiology lab's GeneXpert procedures have been externally validated by the Ugandan Ministry of Health as well as internally validated via Cepheid QA/QC procedures ([67]). GeneXpert testing will be utilized to test for CT, NG and TV.

Number of samples: Objective STI testing will be offered to up to 150 women (this number will vary in light of our study currently in enrolment).

All women will either self-collect or undergo nurse-collected vaginal swabs (2 swabs each) at study initiation, and either at 6 months or at the time of study exit prior to 6 months. Women with STI symptoms at the 3 month study visit will also undergo additional vaginal swab testing, and given our knowledge of symptomatic STIs, we anticipate this to be no larger than 15% of our study population.[68] Therefore we anticipate collecting no more than 645 vaginal swabs in total. The second vaginal swab collected at each instance will be used for quality assurance or stored for future testing.

Use: Vaginal swabs will be collected and analyzed at our corresponding laboratory to test for Chlamydia trachomatis, Neisseria gonorrhoeae, and Trichomonas vaginalis via GeneXpert rapid nucleic acid amplification testing (NAAT).

Collection and sample preparation: Vaginal swabs will be collected via 2 plastic dacron swab, placed in medium, and then stored at 2 to 8 degrees Celsius until the lab is able to perform testing. Samples can be frozen at -20 to -80 degrees for long-term storage.

Shipment and Storage: Vaginal swab samples will be analyzed on-site at the MUST Microbiology Lab. Long-term storage of the secondary swab will be necessary for a proportion of these samples to undergo a secondary confirmatory test for validation of our a subset of the lab results. These materials may also be used by other researchers involved with this study who, in the future, may think of other additional tests, related to our research questions pertaining to HIV risk, PrEP use and STIs, that they would like to perform on the remaining vaginal swabs within 10 years of collection. Before we proceed with any future tests, we will submit the required proposals for ethics review by the university ethics review committee and the national council of science and technology.

STI Blood Testing (MUST Research Lab):

Number of samples: As stated above for vaginal swabbing, we will offer STI testing for up to 150 participants to undergo objective STI testing. If we assume 15% of patients will

have STI symptoms,[68] we would then anticipate having no more than 322 samples of blood.

Use: 10cc of whole blood will be drawn from each study participant to test for HSV2 Ab and Syphilis Treponemal Ab. If the Syphilis Treponemal Ab is positive, this test will automatically reflex to a non-treponemal test to confirm the diagnosis and elucidate whether this is a new or old infection.

Collection and sample preparation: Blood samples should be held for approximately 15 minutes until a clot forms and should then be centrifuged to separate the serum from the blood clot. Samples can be stored at 2 to 8 degrees Celsius for up to five days or frozen at -20 to -80 degrees for long-term storage.

Shipment and Storage: Blood samples for Syphilis will be analyzed on-site. Blood samples for HSV2 Ab will be preprocessed and temporarily stored at -80C for final testing at Makerere University in Kampala (see above). No long-term storage is necessary for these samples.

VII. REIMBURSEMENTS

Participants will receive a transportation refund for all study visits. This reimbursement will be determined based on the distance from the participant's home to the clinic, and can range from 10,000 UGX to 30,000 UGX. Additionally, participants will receive an incentive of 20000 Ush to compensate them for their time.

VIII. RISKS AND DISCOMFORTS:

The potential risks incurred by participating in the studies include:

1. Risk of acquisition of HIV: Based on inclusion criteria, all participants will be at risk for acquiring HIV. It is therefore possible that some participants will become infected over the course of the follow-up study. All participants will be offered comprehensive counselling regarding HIV risks as well as safer conception risk. Participants will be counselled about and able to access condoms in the clinic. Women who seroconvert will be offered comprehensive HIV care at the ISS clinic.
2. Risk of developing resistant virus: Women who become acutely infected with HIV while incompletely taking TDF/FTC PrEP have an increased chance of developing resistant virus. Quarterly adherence counselling sessions will emphasize that women should come to the clinic for additional testing should she develop symptoms of acute HIV infection. Furthermore, if women adhere well, the risk of getting HIV and therefore of getting resistant virus is low.
3. Risk of partner related violence: We will promote serostatus disclosure between partners as part of our safer conception strategy. Discovery of HIV-serodiscordant status may be associated with harm to women. In addition, women who do not disclose PrEP use, may be at risk for partner-related violence should her use of this drug be discovered, given the implications that she therefore perceives herself to be at risk for HIV acquisition. In order to reduce the chance of partner violence we will facilitate referrals to couples-based HIV counselling and testing in the same space as the research is conducted.
4. Risk of Adverse effects related to taking TDF/FTC PrEP: TDF/FTC is first-line therapy in the treatment of HIV. In patients taking TDF/FTC with other HIV medicines to treat HIV infection, common side effects include: diarrhea, nausea, tiredness, headache, dizziness, depression, problems sleeping, abnormal dreams, and rash. In clinical trials of TDF/FTC as PrEP in which most people took study drug, side effects were reported by about 10% of participants (including those in the placebo group). In the Partners

PrEP study group the most common reported symptoms was headache reported by 16% in each group (TDF, TDF/FTC, and placebo.) Other risks reported with TDF/FTC exposure include: gastrointestinal intolerance, such as nausea, diarrhea or vomiting, and flatulence. Rare but serious side effects (not reported in PrEP trials to date) include lactic acidosis/ severe hepatomegaly with steatosis, renal impairment, including cases of acute renal failure and Fanconi's syndrome (renal tubular injury with severe hypophosphatemia), increase in bone metabolism leading to osteopenia, hypersensitivity reaction, acute exacerbation of hepatitis B infection, body fat redistribution, and risk of drug resistance in individuals with undiagnosed HIV. Women will be expressly counselled on the risks and benefits of PrEP during both the periconception and pregnancy period according to CDC and WHO guidelines. Women will be closely monitored in study visits and complete symptom logs. A clinician will be a part of the study staff and PrEP will be held or discontinued for women experiencing adverse effects based on his/her clinical judgment. Data on adverse events and side effects will be reported to the Antiretroviral in Pregnancy Registry.

5. Risk of Stigma: There is a potential that enrolled participants will be stigmatized if they reveal their involvement with the study or become associated with the study in some other way.
6. Social harms and psychological stress: Social harm, including emotional distress (e.g., stigma, compromise of reproductive autonomy), is a potential risk for participants. Another potential risk for participants is psychological harm, where the participant may possibly behave in a manner indicating distress (crying, showing aggression) or verbally expresses anxiety, fear or anger regarding own and or a partner's HIV serostatus or new STI diagnoses, interpersonal and or abuse issues within their partnership, pregnancy and or HIV serostatus disclosure concerns, as well as concerns with PrEP initiation and or adherence. Should this occur the protocol for managing participant distress will be implemented. The study team member will immediately stop the study procedures and talk with the participant about referral to a counsellor for support. Distressed participants will be referred to the local clinic for psychological counselling as per the routine standard of care at the clinic (please see Participant Distress Referral Form). After some time is allowed for recovery, the study team member will follow up with the participant to check their progress.
7. Risk to confidentiality: Risk to confidentiality exist in all studies. To protect against breach of confidentiality, participants will be assigned a unique study identifier, participant names will not be used in any recorded interviews or paper forms, all documents will be kept in locked cabinets in the study site office in the ISS clinic in Mbarara. All other study data will be associated with participants' coded identification number only. A master list of participant names and their code numbers will be stored in a locked file cabinet separate from the rest of the data. Only Dr. Matthews, Dr, Bosco and the study coordinator will have access to the list, which will be destroyed within two years of publication of study findings, or if there is no publication, no later than six years after the study has ended. All interviews will be conducted in private locations at the research site. Safer conception counselling and adherence counselling will occur in individual sessions with a trained counsellor. All data will be kept confidential and stored in locked cabinets within restricted areas of MUST. All key drives and computers will be password protected. Sexual behaviour data collected by SMS will be protected in the servers and the messages themselves will be password protected.
8. Risk of time inconvenience: There may be a time inconvenience to study participants but the study staff will work with participants to reduce study visit burden while still maintaining the integrity of the research aims.

9. Risk of discomfort: There is a risk that participants may feel uncomfortable discussing issues related to HIV status, STI history, fertility desires and other emotionally-charged or personal subjects during visits. However, we do not wish for this to happen, and participants may choose not to answer if talking about these issues makes them uncomfortable. On-site counselling services will be made available for participants who express distress. In addition to psychological discomfort, participation in blood draws for testing may result in physical discomfort including but not limited to: faintness, inflammation of the vein, pain, bruising and/or bleeding at the puncture site. There is also a small possibility of infection. The physical discomfort from blood draw is unlikely to result in any serious adverse reaction but may cause irritation at the puncture site for several days. In order to best protect participants from potential discomforts phlebotomy procedures for biological and adherence testing will be performed by experienced personnel under sterile conditions. Vaginal swabs can also cause some discomfort both physically and potentially psychologically, but this will be minimized by encouraging all women to self-collect vaginal swabs.
10. Unforeseen Risks: The study medicine is investigational for use as PrEP (but approved for treatment of HIV) in Uganda. It is approved for use as PrEP in the U.S. and Europe and recommended by the W.H.O. However, there may be unforeseen or unknown risks or side effects. Women will be expressly counselled on the potential for unforeseen risks before initiating or continuing TDF/FTC as PrEP during periconception. In addition, women will be counselled on how and when to contact the study clinician if they experience any additional or potentially unforeseen side effects throughout their study participation. Given the data acquired to date in large clinical trials and demonstration projects of TDF/FTC as PrEP and the decades' experience using TDF/FTC for the treatment of HIV, there is no reason to expect that the drug will not work as intended.

IX. POTENTIAL BENEFITS

Potential benefits to participants: The participant may benefit from the information gathered from this study by learning strategies for reducing periconception HIV transmission. Participants who choose to initiate and adhere to safer conception strategies will also be engaging in HIV-risk reduction. PrEP and safer conception education are not routinely offered in the public health sector. Women participating in the study may have a reduced risk of HIV transmission by virtue of their participation. Evaluating uptake of and adherence to antiretrovirals as pre-exposure prophylaxis in this population is crucial to understanding whether and how this prevention strategy should be incorporated into HIV-risk reduction packages for at-risk women planning or with pregnancy. Women will also benefit from receiving objective STI testing to achieve an accurate diagnosis and treatment for their STIs which will result in decreased morbidity for the participant and her potential children. In addition, participants will have access to regular testing and referrals to ongoing care.

Potential benefits to society (e.g., increased understanding of disease processes): Women who choose to conceive with an infected or unknown serostatus partner in HIV-endemic settings need prevention strategies to reduce periconception HIV acquisition risk. Women at high risk for acquiring HIV during pregnancy also need risk reduction strategies to protect themselves and their babies. Evaluating uptake of and adherence to antiretrovirals as pre-exposure prophylaxis is crucial to understanding whether and how this prevention strategy should be incorporated into HIV risk reduction packages for at risk women planning or with pregnancy. Many PrEP studies focused on men who have sex with men (MSM) have shown that patients have an increase in

STIs.[69, 70] Understanding the STI risk among women, especially in the periconception period is a crucial first step in the process of designing PrEP programs and understanding patient risk in regards to HIV and STIs.

X. ALTERNATIVES TO PARTICIPATION

Participation is completely voluntary. Deciding not to participate will not have any bearing on the participant's ability to access clinical existing clinical services.

XI. SOURCES OF ADDITIONAL INFORMATION

For the duration of the study, enrolled women will be under the care of Dr. Mwebesa Bosco Bwana who is the clinician for the study. Women will be expressly counselled on when and how to contact Dr. Mwebesa Bosco Bwana throughout the duration of their participation. Dr. Mwebesa Bosco Bwana and other study staff will be available to participants between their study visits to discuss potential symptoms or problems.

XII. MANAGEMENT OF ADVERSE EVENTS:

Adverse events and serious adverse events will be assessed and logged by study staff at each study visit. Unanticipated adverse events reported to the clinic staff or study clinician outside of normal study visits will be recorded and participants will be referred to appropriate care. All adverse events will be reviewed by the Principal Investigators and other relevant study/clinical staff and reported to appropriate study ethics committees (MUST IRB, Partners Healthcare IRB).

XIII. POST STUDY ACCESS TO TDF/FTC

PrEP is not currently provided in the public sector in Uganda. We will inform exiting participants about their options for accessing it in the private sector, or, should it become available, in the public sector.

XIV. BIOSTATISTICAL ANALYSIS

Analysis, Aim 1

Hypothesis: 70% of women will initiate PrEP as part of a safer conception strategy. In the Partners PrEP demonstration project, PrEP uptake is ~90% among uninfected women in mutually-disclosed serodiscordant partnerships (JM Baeten, personal communication). We expect uptake to be lower among women who are not in mutually-disclosed serodiscordant partnerships and who may worry about the effects of PrEP on pregnancy. We hypothesize that PrEP uptake will be associated with relationship power and social support.

Table 4. 95% CIs for range of uptake and adherence.

% uptake or adherence	Uptake, N = 150 95% CI	Adherence[1], N = 105 95% CI
50	42.5-57.5%	40.4-59.6%
60	52.6-67.4%	50.6-69.4%
70	63.1-76.9%	61.2-78.8%
80	74.0-86.0%	72.3-87.7%
90	85.5-94.5%	84.3-95.7%

1. Detectable drug at visit 3 months after initiation

Estimates of PrEP uptake: PrEP uptake/initiation is defined as the proportion of enrolled women who collect at least one 3-month supply of PrEP.A target sample size of 150 women produces a 95% CI width between 13% (when 70% initiate PrEP) and 15% (when 50% initiate PrEP) (Table 4.) We will calculate the prevalence (and 95% CI) of PrEP initiation using an intercept-only logistic regression model to account for potential clustering within the two groups (women enrolled through partners, women enrolled as individuals). We will describe, compare and test for differences between baseline characteristics of women who do and do not initiate PrEP. Log binomial regression with GEE will be used to estimate univariable and multivariable-adjusted risk ratios (RR) and 95% CIs for the associations between relationship power/social support and PrEP initiation. We will assess potential confounding factors individually; any factor with P≥0.20 will be excluded from further consideration and the remaining covariates entered into a multivariable model.

Analysis, Aim 2

Hypothesis: 70% of women will have detectable drug 3 months after PrEP initiation.

Estimates of periconception PrEP adherence: Periconception PrEP adherence will be defined as PrEP initiators with plasma TFV concentration ≥ 40ng/mL three months after initiating PrEP. A target sample size of 105 women initiating PrEP will provide 95% CI width of 18% when the percent of high adherers is 80% (Table 4). Secondary adherence outcomes are based on Wisepill device monitoring. We will determine the mean and median number of pills taken as well as patterns of adherence (gaps). We will consider Wisepill adherence as a binary outcome defined as taking ≥ 80% of prescribed doses based on biologic plausibility and prior studies [23, 65, 71, 72]. Monthly assessments of median (interquartile range) adherence and number of 72-hour adherence gaps will be used to examine longitudinal trends in adherence.

Table 5: Minimal detectable RR at 80% power, alpha 0.5, comparing women with and without exposure characteristic, varying distribution of exposure, varying percentages of PrEP uptake / adherence.

% with high relationship power	% initiatingPrEP in unexposed group (n = 150)	Min detectable RR	% adherent in unexposed group (n = 105)	Min detectable RR
20%	30%	1.94	30%	2.14
	50%	1.53	50%	1.63
	70%	1.30	70%	1.35
	80%	1.21	80%	1.24
40%	30%	1.77	80%	1.24
	50%	1.45	30%	1.94
	70%	1.27	50%	1.54
	80%	1.19	80%	1.22
60%	30%	1.76	70%	1.32
	50%	1.46	80%	1.22
	70%	1.28	30%	1.94
	80%	1.20	80%	1.23

We will calculate prevalence (and 95%CI) of PrEP adherence using an intercept-only logistic regression model with GEE. Log binomial regression with GEE will be used to estimate univariable and multivariable-adjusted risk ratios (RR) and 95% CIs for the associations between relationship power/social support and PrEP adherence.

We will assess potential confounding factors individually; any factor with P≥0.20 will be excluded from further consideration and the remaining covariates entered into a multivariable model. For continuous adherence outcomes, we will compare mean and median adherence levels across the periconception period according to levels of relationship power and social support. Depending on the adherence data distribution, we will fit univariable and multivariable linear or log-normal regression models with GEE. For the number of 72-hour adherence gaps, we will fit Poisson regression models with GEE. Table 5 shows the minimum differences we expect to detect with 80% power given a range of PrEP uptake/adherence and a range of distributions of exposures, where exposure to a certain characteristic elevates the prevalence of PrEP uptake/adherence. For example, to evaluate the association of relationship power on PrEP initiation, if the background prevalence of high relationship power is 20% the background prevalence of PrEP initiation among women with low relationship power is 50%, a sample size of 150 provides 80% power to detect a 53% increased likelihood of PrEP initiation (RR=1.53) among those with higher relationship power.

We will describe uptake of and adherence to other safer conception strategies (e.g. proportion who time condomless sex to peak fertility, who delay condomless sex until partner is on ART), pregnancy incidence, and HIV seroconversion (and compare rates among those taking and adherent to PrEP with those who do not take or adhere to PrEP). All power and sample size calculations were calculated using Pass II software.

Analysis, Aim 3

We will use grounded theory methods to analyze interview data. These methods lay out a systematic set of inductive procedures for developing categories of information, organizing data in terms of these categories through coding, connecting the categories, and deriving from these connections an empirically grounded theory[73].

Analysis, Aim 4

Aim 4.1. We will measure prevalence of STIs in up to 150 HIV-negative women who want to conceive a child while at risk for HIV exposure.

Aim 4.2. We will measure six-month incidence of STIs in up to 150 HIV-negative women who conceive or want to conceive a child while at risk for HIV exposure.

Hypothesis 4.2. We expect a higher incidence of condomless sex leading to a higher STI incidence among women who choose PrEP as part of a safer conception strategy compared to women who do not take PrEP.

We intend to evaluate up to 150 HIV-negative women entering the study and estimate the prevalence of any STI and each of the specific STIs. Based on prior work, we anticipate the incidence of any STI will be approximately 35%[74] and that 2 out of 3 women[75] will want to start PrEP. We would have 82% power for a difference of 43% (PrEP group) vs 20% (no PrEP group) using a Chi-square test (alpha=0.05, two-sided).

For Aim 4.1, we will use logistic regression first to test covariates as predictors of having a STI at baseline and then to develop an initial model predicting STI. This model will be applied to the STI incidence data through six months to assess operating characteristics. For Aim 4.2, we will use a logistic regression model predicting incident STIs over six months including PrEP in all models and then adding the number of condomless sex acts to the model. If the number of

condomless sex acts is a significant predictor and there is a substantial reduction in the odds ratio (OR) for PrEP when the number of condomless sex acts is included in the model, then we will conclude that this is a mediator of the PrEP effect.

XV. MONITORING AND QUALITY ASSURANCE

The Ugandan PI will be responsible for continuous data and safety monitoring of all study participants and for alerting the US PI if unexpected concerns arise. Serious adverse events (e.g. physical or emotional injury) will be reported to all involved IRBs according to the guidelines of each IRB (i.e., within ten days for the Partners' IRB) and to Gilead who will be providing study drug.

Any protocol violations will be reported within the required timeframe (as per MUST IRB / Partners IRB requirements).

All study-related information will be stored securely at the study site. All participant information will be stored in areas with limited access. Data collection will be labelled only by a participant identification number to maintain participant confidentiality. All records that contain names or other personal identifiers, such as locator forms and informed consent forms, will be stored separately from study records labelled with the participant identification number. All databases will be secured with password-protected access systems. The master list linking participant name to study identification number and other identifying information in study files will be retained for six years after the study is completed; after that time, the link will be destroyed.

We will review the data bi-annually with particular attention to possible adverse events (including HIV acquisition, medication-related adverse events, adverse pregnancy outcomes, and social harms) among women participating in this study with a focus on:

- Rate of HIV seroconversion, compared against the anticipated rate of incident HIV infection in the study population.
- Serious adverse events and medication side effects.
- Social harm, including emotional distress (e.g., stigma, compromise of reproductive autonomy).
- Study execution, including enrolment rates, retention, PrEP initiation, and adherence.

XVI. Budget for MUST

	Year 1	Year 2	Year 3
	7/1/16-6/30/17	7/1/17-6/30/18	7/1/18-6/30/19
Personnel	$74,766	$74,766	$74,766
Consultants	$0	$0	$0
STI laboratory fees	$0	$2,000	$12,714
Equipment	n/a	n/a	n/a
Supplies	$850	$150	$0
STI testing supplies	$0	$100	$430
STI shipping fees for confirmatory testing	$0	$200	$800

STI treatment medications	$0	$50	$295
Travel	$2,846	$4,346	$5,862
Subject Costs	$7,500	$7,500	$0
Other	$64,038	$63,238	$69,372
**Direct Costs**	**$150,000**	**$150,000**	**$150,000**
**Indirect Costs**	**$15,000**	**$15,000**	**$15,000**
**TOTAL**	**$165,000**	**$165,000**	**$165,000**

REFERENCES

1. The_World_Bank. *Fertility rate (total births per woman), Table*. 2013 12 September 2013]; Word Development Indicators]. Available from: http://data.worldbank.org/indicator/SP.DYN.TFRT.IN.
2. Ministry_of_Health, U. and UNAIDS, *HIV and AIDS Uganda Country Progress Report 2014.* 2015.
3. Beyeza-Kashesya, J., et al., *My partner wants a child: a cross-sectional study of the determinants of the desire for children among mutually disclosed sero-discordant couples receiving care in Uganda.* BMC Public Health, 2010. **10**: p. 247.
4. Heys, J., et al., *Fertility desires and infection with the HIV: results from a survey in rural Uganda.* AIDS, 2009. **23 Suppl 1**: p. S37-45.
5. Nakayiwa, S., et al., *Desire for children and pregnancy risk behavior among HIV-infected men and women in Uganda.* AIDS Behav, 2006. **10**(4 Suppl): p. S95-104.
6. Wanyenze, R.K., et al., *Fertility and contraceptive decision-making and support for HIV infected individuals: client and provider experiences and perceptions at two HIV clinics in Uganda.* BMC Public Health, 2013. **13**: p. 98.
7. Wagner, G. and R.K. Wanyenze, *Fertility desires and intentions and the relationship to consistent condom use and provider communication regarding childbearing among HIV clients in Uganda.* ISRN Infectious Diseases, 2013.
8. Case, K.K., et al., *Understanding the modes of transmission model of new HIV infection and its use in prevention planning.* Bull World Health Organ, 2012. **90**(11): p. 831-838A.
9. Beyeza-Kashesya, J., et al., *The dilemma of safe sex and having children: challenges facing HIV sero-discordant couples in Uganda.* African Health Sciences, 2009. **9**(1): p. 2-12.
10. Kastner, J., et al., *Antiretroviral Therapy Helps HIV-Positive Women Navigate Social Expectations for and Clinical Recommendations against Childbearing in Uganda.* AIDS Res Treat, 2014. **2014**: p. 626120.
11. Matthews, L.T., et al., *"I always worry about what might happen ahead": Implementing safer conception services in the current environment of reproductive counseling for HIV-affected men and women in Uganda.* BioMed Central: International Research, in press, 2015.
12. Matthews, L.T., et al., *Antiretrovirals and safer conception for HIV-serodiscordant couples.* Curr Opin HIV AIDS, 2012. **7**(6): p. 569-578.
13. Bekker, L.G., et al., *Guideline on safer conception in fertile HIV-infected individuals and couples.* The Southern African Journal of HIV Medicine, 2011. **12**(2): p. 31-44.
14. Eke, A.C. and C. Oragwu, *Sperm washing to prevent HIV transmission from HIV-infected men but allowing conception in sero-discordant couples.* Cochrane Database Syst Rev, 2011(1): p. CD008498.
15. Matthews, L.T., et al., *Consistently high study medication adherence during periconception periods among HIV-1-uninfected women participating in a clinical trial of antiretroviral pre-exposure prophylaxis.* JAIDS, 2014. **67**(1): p. 91-97.
16. Matthews, L.T., et al., *Periconception pre-exposure prophylaxis to prevent HIV transmission: benefits, risks, and challenges to implementation.* AIDS, 2010. **24**(13): p. 1975-1982.
17. Cohen, M.S., et al., *Prevention of HIV-1 infection with early antiretroviral therapy.* N Engl J Med, 2011. **365**(6): p. 493-505.
18. Grosskurth, H., et al., *Impact of improved treatment of sexually transmitted diseases on HIV infection in rural Tanzania: randomised controlled trial.* Lancet, 1995. **346**(8974): p. 530-6.
19. Wawer, M.J., et al., *A randomized, community trial of intensive sexually transmitted disease control for AIDS prevention, Rakai, Uganda.* Aids, 1998. **12**(10): p. 1211-25.

20. Semprini, A.E., et al., *Insemination of HIV-negative women with processed semen of HIV-positive partners.* Lancet, 1992. **340**(8831): p. 1317-9.
21. Weller, S. and K. Davis, *Condom effectiveness in reducing heterosexual HIV transmission.* Cochrane Database Syst Rev, 2002(1): p. CD003255.
22. World_Health_Organization, *Guidance on couples HIV testing and counselling including antiretroviral therapy for treatment and prevention in serodiscordant couples*. 2012: Geneva.
23. Baeten, J.M., et al., *Antiretroviral Prophylaxis for HIV Prevention in Heterosexual Men and Women.* N Engl J Med, 2012. **367**(5): p. 399-410.
24. Thigpen, M.C., et al., *Antiretroviral Preexposure Prophylaxis for Heterosexual HIV Transmission in Botswana.* N Engl J Med, 2012. **367**(5): p. 423-34.
25. Donnell, D., et al., *HIV protective efficacy and correlates of tenofovir blood concentrations in a clinical trial of PrEP for HIV prevention.* J Acquir Immune Defic Syndr, 2014. **66**(3): p. 340-8.
26. Ministry_of_Health, U. and UNAIDS, *HIV and AIDS Uganda Country Progress Report.* 2014.
27. Uganda_Bureau_of_Statistics and ICF_International_Inc., *Uganda Demographic and Health Survey 2011*, M.I.I. UBOS and Calverton, Editor. 2012: Kampala, Uganda.
28. Sauer, M.V., et al., *Providing fertility care to men seropositive for human immunodeficiency virus: reviewing 10 years of experience and 420 consecutive cycles of in vitro fertilization and intracytoplasmic sperm injection.* Fertil Steril, 2009. **91**(6): p. 2455-60.
29. Centers_for_Disease_Control. *Elimination of Mother-to-Child Transmission (EMTCT) in the United States*. 2014 [cited 2015 December 22, 2015]; Available from: http://www.cdc.gov/hiv/group/gender/pregnantwomen/emct.html.
30. Panel_on_Treatment_of_HIV-Infected_Pregnant_Women_and_Prevention_of_Perinatal_Transmission, *Recommendations for use of antiretroviral drugs in pregnant HIV-1-infected women for maternal health and interventions to reduce perinatal HIV transmission in the United States*. 2014.
31. Schwartz, S.R., et al., *Implementation of a safer conception service for HIV-affected couples in South Africa.* AIDS, 2014. **28 Suppl 3**: p. S277-85.
32. Pintye, J., et al., *Fertility Decision-Making Among Kenyan HIV-Serodiscordant Couples Who Recently Conceived: Implications for Safer Conception Planning.* AIDS Patient Care STDS, 2015. **29**(9): p. 510-6.
33. Marrazzo, J.M., et al., *Tenofovir-based preexposure prophylaxis for HIV infection among African women.* N Engl J Med, 2015. **372**(6): p. 509-18.
34. Van Damme, L., et al., *Preexposure Prophylaxis for HIV Infection among African Women.* N Engl J Med, 2012. **367**(5): p. 411-22.
35. Bekker, L.G., et al. *HPTN 067/ADAPT Cape Town: A comparison of daily and nondaily*

PrEP dosing in African women, poster 978LB. in *CROI*. 2015. Seattle.

36. van der Straten, A., et al., *Women's experiences with oral and vaginal pre-exposure prophylaxis: the VOICE-C qualitative study in Johannesburg, South Africa.* PLoS One, 2014. **9**(2): p. e89118.
37. Corneli, A., et al., *Perception of HIV Risk and Adherence to a Daily, Investigational Pill for HIV Prevention in FEM-PrEP.* J Acquir Immune Defic Syndr, 2014. **67**(5): p. 555-63.
38. World_Health_Organization, *Guideline on when to start antiretroviral therapy and on pre-exposure prophylaxis for HIV*. 2015, W.H.O.: Geneva.
39. Lampe, M.A., et al., *Achieving safe conception in HIV-discordant couples: the potential role of oral preexposure prophylaxis (PrEP) in the United States.* Am J Obstet Gynecol, 2011. **204**(6): p. e1-e8.
40. Whetham, J., et al., *Pre-exposure prophylaxis for conception (PrEP-C) as a risk reduction strategy in HIV-positive men and HIV-negative women in the UK.* AIDS Care, 2013.

41. Vernazza, P.L., et al., *Preexposure prophylaxis and timed intercourse for HIV-discordant couples willing to conceive a child.* AIDS, 2011. **25**(16): p. 2005-8.
42. U.S._Public_Health_Service, Centers_for_Disease_Control_and_Prevention, and U. Department_of_Health_and_Human_Services, *Preexposure prophylaxis for the prevention of HIV infection in the United States - 2014. Clinical Providers' Supplement.* 2014.
43. Carey, M.P. and K.E. Schroder, *Development and psychometric evaluation of the brief HIV Knowledge Questionnaire.* AIDS Educ Prev, 2002. **14**(2): p. 172-82.
44. Matthews, L.T., et al., *Periconception HIV risk behavior among men and women reporting HIV-serodiscordant partnesr in KwaZulu-Natal, South Africa.* AIDS and Behavior, 2015.
45. Napper, L.E., D.G. Fisher, and G.L. Reynolds, *Development of the perceived risk of HIV scale.* AIDS Behav, 2012. **16**(4): p. 1075-83.
46. Kalichman, S.C., *Post-exposure prophylaxis for HIV infection in gay and bisexual men. Implications for the future of HIV prevention.* Am J Prev Med, 1998. **15**(2): p. 120-7.
47. van Balen F, T.-K.T., *Involuntarily childless couples: their desire to have children and their motives. .* J Psychosom Obstet Gynaecol 1995. **16**: p. 137-144.
48. Tsai, A.C., et al., *Internalized stigma, social distance, and disclosure of HIV seropositivity in rural Uganda.* Ann Behav Med, 2013. **46**(3): p. 285-94.
49. Stirratt, M.J., et al., *The role of HIV serostatus disclosure in antiretroviral medication adherence.* AIDS Behav, 2006. **10**(5): p. 483-493.
50. Pulerwitz, J., S.L. Gortmaker, and W. DeJong, *Measuring sexual relationship power in HIV/STD research.* Sex Roles, 2000. **42**(7/8): p. 637-620.
51. Milhausen, R.R., Sales, J.M., Wingood, G.M., DiClemente, R.J., Salazar, L.F., & Crosby, R.A, *Validation of a Partner Sexual Communication Scale for Use in HIV/AIDS Prevention Interventions.* Journal of HIV/AIDS Prevention in Children & Youth, 2007. **8**: p. 11 – 33.
52. Broadhead, W.E., *The Duke-UNC Functional Social Support Questionnaire. Measurement of social support in family medicine patients. .* MEDICAL CARE, 1998. **26**(7): p. 709-23.
53. Visser, M.J., et al., *Development of parallel scales to measure HIV-related stigma.* AIDS Behav, 2008. **12**(5): p. 759-71.
54. Crankshaw, T.L., et al., *A conceptual framework for periconception HIV transmission risk reduction among HIV serodiscordant couples.* Reproductive Health Matters, 2012. **20**(39): p. 50-60.
55. Bunting, L. and J. Boivin, *Development and preliminary validation of the fertility status awareness tool: FertiSTAT.* Hum Reprod, 2010. **25**(7): p. 1722-33.
56. *PRAMS model surveillance protocol*. 2009 [cited 2009; Available from: http://www.cdc.gov/prams.
57. Ahluwalia, I.B., et al., *Pregnancy Risk Assessment Monitoring System (PRAMS): unintended pregnancy among women having a live birth. PRAMS Working Group.* J Womens Health Gend Based Med, 1999. **8**(5): p. 587-9.
58. Klerman, L.V., *The intendedness of pregnancy: a concept in transition.* Matern Child Health J, 2000. **4**(3): p. 155-62.
59. Rogers, M.M., I.B. Ahluwalia, and C.L. Melvin, *The pregnancy risk assessment monitoring system (PRAMS).* J Womens Health, 1998. **7**(7): p. 799-801.
60. Mudau, M., et al., *High prevalence of asymptomatic sexually transmitted infections among human immunodeficiency virus-infected pregnant women in a low-income South African community.* Int. J. STD AIDS, 2017: p. 956462417724908.
61. Alam, N., et al., *Partner notification for sexually transmitted infections in developing countries: a systematic review.* BMC public health, 2010. **10**(1): p. 19.

62. Hendrix, C.W., et al., *Dose Frequency Ranging Pharmacokinetic Study of Tenofovir-Emtricitabine After Directly Observed Dosing in Healthy Volunteers to Establish Adherence Benchmarks (HPTN 066).* AIDS Res Hum Retroviruses, 2016. **32**(1): p. 32-43.
63. Castillo-Mancilla, J.R., et al., *Short communication: Tenofovir diphosphate in dried blood spots as an objective measure of adherence in HIV-infected women.* AIDS Res Hum Retroviruses, 2015. **31**(4): p. 428-32.
64. Liu, A., et al., *Patterns and correlates of PrEP drug detection among MSM and transgender women in the Global iPrEx Study.* J Acquir Immune Defic Syndr, 2014. **67**(5): p. 528-37.
65. Haberer, J.E., et al., *Adherence to Antiretroviral Prophylaxis for HIV Prevention: A Substudy Cohort within a Clinical Trial of Serodiscordant Couples in East Africa.* PLoS Med, 2013. **10**(9): p. e1001511.
66. Lingappa, J., et al., *Sensitivity and specificity of herpes simplex virus-2 serological assays among HIV-infected and uninfected urban Ugandans.* International journal of STD & AIDS, 2010. **21**(9): p. 611-616.
67. Bahr, N.C., et al., *Diagnostic accuracy of Xpert MTB/RIF Ultra for tuberculous meningitis in HIV-infected adults: a prospective cohort study.* Lancet Infect Dis, 2018. **18**(1): p. 68-75.
68. Mlisana, K., et al., *Symptomatic vaginal discharge is a poor predictor of sexually transmitted infections and genital tract inflammation in high-risk women in South Africa.* J. Infect. Dis., 2012. **206**(1): p. 6-14.
69. Volk, J.E., et al., *No New HIV Infections With Increasing Use of HIV Preexposure Prophylaxis in a Clinical Practice Setting.* Clin. Infect. Dis., 2015. **61**(10): p. 1601-1603.
70. Liu, A.Y., et al., *Preexposure Prophylaxis for HIV Infection Integrated With Municipal- and Community-Based Sexual Health Services.* JAMA Intern. Med., 2016. **176**(1): p. 75-84.
71. Garcia-Lerma, J.G., et al., *Prevention of rectal SHIV transmission in macaques by daily or intermittent prophylaxis with emtricitabine and tenofovir.* PLoS Med, 2008. **5**(2): p. e28.
72. Grant, R.M., et al., *Preexposure Chemoprophylaxis for HIV Prevention in Men Who Have Sex with Men.* N Engl J Med, 2010. **363**(27): p. 2587-99.
73. Miles, M.B. and A.M. Huberman, *Qualitative Data Analysis: An expanded sourcebook*. 2nd ed. 1994, Thousand Oaks, CA: SAGE Publications, Inc.
74. Kaida, A., et al. *High burden of asymptomatic genital tract infections among sexually active South African youth: Considerations for HIV prevention programs.* in *Poster presented at: South African AIDS Conference*. June 2017. Durban, South Africa.
75. Matthews, L.T., et al., *High Medication Adherence During Periconception Periods Among Hiv-1–uninfected Women Participating in a Clinical Trial of Antiretroviral Pre-exposure Prophylaxis.* Jaids Journal of Acquired Immune Deficiency Syndromes, 2014. **67**(1): p. 91-97.

XVII.
