# Supplementary material for: High PrEP uptake and objective longitudinal adherence among HIV-exposed women with personal or partner plans for pregnancy in rural Uganda: A cohort study
Source: PLoS Med. 2023 Feb 16;20(2):e1004088. doi: 10.1371/journal.pmed.1004088 (PMC9983833; doi:10.1371/journal.pmed.1004088)
Supplement: Attachment — Submitted filename: Response to Review Final.docx [file pmed.1004088.s007.docx]

**RESPONSE TO REVIEW**

**Comments from the Academic Editor:**

In particular, I would like to see the authors respond to:

- Reviewer 1 point 8 (representativeness/generalizability).
- Finally, Reviewer 1's point about the representativeness of this population seems important. The large proportion of potential participants who screened out because they did not want a baby (more than half those screened, Figure 2) seems a bit at odds with the claims about high fertility in this population and makes me wonder if targeting of this intervention is needed.

**Author Response: Thank you for this observation. We have addressed this concern in the 3^rd^ paragraph of the discussion, which now reads:**

**“Our screen to enroll ratio was high (916:131, 0.14), and may raise questions about the generalizability of the intervention. Over half of those excluded denied personal or partner pregnancy in the next year. Unfortunately, people who want to have children while exposed to HIV often face internalized, community, and healthcare stigmas around their choices given the risk of perinatal HIV acquisition [75-78]. Admitting being in an HIV-serodifferent partnership can also lead to stigma. Thus, some of the eligible people may have screened out due to social desirability bias. In addition, most of our recruitment occurred through HIV testing and counseling sites. Further reach into the community and non-HIV focused clinical settings could enhance generalizability in future iterations. Finally, many women in pro-natal settings like Uganda do not plan for pregnancy and this may have led to limitations in recruitment. In future iterations of this work, we hope to adapt the intervention to also meet the HIV prevention needs of women with pregnancy and post-partum, thus expanding the reach and potential impact of the intervention. As opportunities to prevent HIV in the context of reproductive goals become more common, stigma may reduce.”**

- Reviewer 2 point 4 and Reviewer 3 (transmission despite high adherence)

**Author Response: Adherence inherently involves a degree of uncertainty, and reliance or confidence in a single measure can lead to misinterpretation. The apparent inaccuracy of electronic pillbox adherence with the one participant who acquired HIV does not invalidate the measure, which has been useful in understanding patterns of adherence (e.g., Haberer, JAIDS, 2021; Musinguzi, AIDS Behav 2022). We have strengthened the discussion on this point and again highlighted that a strength of the manuscript is using several different measures of adherence.**

**“Differences observed across the PrEP adherence measures in this cohort highlight the value of incorporating multiple adherence measurements and expose some gaps in our understandings of tenofovir drug-level data interpretation for cis-gender women. Importantly, adherence by any one of these measures is higher than seen in many prior cohorts of women of reproductive age. Further, one HIV seroconversion was observed despite high pregnancy and STI incidence indicating that the intervention supported women to avoid HIV transmission over time [85]. Exploring possible reasons for discrepancies across measures is important for interpreting the effect of our intervention and informing future analyses. First, the categories of high are not equivalent across the measures in terms of dosing or time period: 80% of pills taken by electronic pillcap equates to 5-6 doses per week averaged over ~90 days, >40ng/mL represents dosing in the last 24 hours, and >600 fmol/punch TFV-DP represents 4 or more doses per week over 6-8 weeks. In addition, the samples represent different groups: pillcap data were available for 83%, plasma data were processed for 39%, and whole blood samples were processed for 93% of women accessing PrEP at the 3-month (primary outcome) timepoint. High adherence by electronic device persisted whereas the proportion with high levels waned over time with plasma and whole blood measures. Women may have persisted using the pillcap device without taking pills to please the researchers or counselors (social desirability bias)[86]. However, studies have shown that adhering to pillcap opening without taking medication is difficult to maintain over time[87]; further, PrEP use was not required to remain in the study. Plasma TFV measures pill-taking in the past week and women may have been prompted to take doses by reminders about upcoming clinic visits thus explaining higher plasma levels even when TFV-DP concentrations were lower; however, correlations between plasma TFV and electronic pillcap data suggest that pillcap use over 30 days aligned with an objective, biologic measure in the week prior to blood draw. Correlations between electronic pillcap and whole blood (DBS) were low. In a secondary analysis of whole blood spots collected from men and women with electronic pillcap data in the Partners PrEP demonstration project, TFV-DP measurements were specific but not sensitive for electronic pillcap adherence [68] with sensitivity lowest for the highest categories of MEMS adherence. Those authors speculate that the discrepancy may relate to interindividual differences in drug disposition [88], [89, 90]. While inter-individual differences in PK are certain, intra-individual PK changes over time are less likely (particularly with respect to anemia, BMI and other biological factors impacting drug disposition.) Thus, the downward trends of DBS TFV-DP and plasma TFV values likely at least partially reflect behavioral fatigue with daily pill taking and highlight the importance of longitudinal adherence support for daily PrEP use. The most conservative estimates of adherence also suggest that some women may require different adherence support than what was provided in this study and ongoing work is needed to optimize adherence support for women [73, 91]. Again, adherence by all measures was higher in this cohort than for most PrEP implementation cohorts among women: the most conservative adherence benchmark is the TFV-dp concentration and 47%, 41%, and 45% of women at months 3, 6, and 9 attained these high levels in this compartment.”**

- Reviewer 4 point 1 (feasibility) and point 2 (measure) and all comments on Discussion.

1. Expanding on one of Reviewer 4's comments, I'd also like to see a much more thorough discussion of the feasibility of scaling up this intervention. Making PrEP available is clearly not sufficient to achieve both high uptake and good adherence. The counseling and support (and perhaps frequent monitoring) that the authors offered must have mattered, but I saw no discussion of this or how such services might be implemented at large scale in routine care. It's not clear whether it would easy or difficult to do so, or what resources are required. I also wonder if the regular study monitoring improved PrEP adherence.

**Author Response: Thank you, we have amended the discussion to now include the following:**

**“The Healthy Families PrEP combination intervention offered counselling delivered by local lay counselors to support HIV prevention strategies including daily PrEP use in the context of planning for or being pregnant (Figure 1). The three counseling sessions offered to all women were developed to be offered to individuals or couples, and we published our qualitative data indicating acceptability and feasibility from the standpoint of clients and providers {Young, 2021 #36}. Quarterly adherence support was adapted from the Lifesteps intervention, an evidence-based intervention included in the CDC compendium of effective interventions for HIV prevention, and utilized education, problem solving and motivational interviewing strategies to promote adherence. Lifesteps was first adapted for PrEP among HIV-serodifferent couples in Uganda and successfully promoted PrEP adherence [46]. Here the same tenets were applied with an added emphasis on the opportunity to prevent HIV acquisition while meeting reproductive goals. We did not collect detailed process data, but counselors were trained to offer sessions lasting 30-60 minutes. Our team provided in-person training and every other month supervision support, where counselors discussed challenges. While this counseling approach was effective for most women in our cohort, some encountered ongoing adherence challenges. Future iterations of this work will aim to identify which women are most likely to experience adherence challenges, and identify what role adherence counseling may play in supporting prevention effective PrEP use, regardless of PrEP formulation (e.g. adaptive interventions [74]). For women who consistently use PrEP, adherence counseling support could be scaled down if desired. A future effectiveness trial and preliminary exploration of implementation strategies in collaboration with the Ugandan Ministry of Health will inform best approaches for public sector implementation and future scaling to meet the needs women of reproductive age. In these next steps, we will evaluate the effectiveness and integration of this approach into routine care to improve PrEP uptake and adherence, including the cost, feasibility, scale-ability and implementation climate/process in Uganda.”**

**Requests from the Editors:**

1. Please update your title to ‘High PrEP uptake and objective longitudinal adherence among HIV-exposed women with personal or partner plans for pregnancy in rural Uganda: a cohort study’

**Author Response: Thank you. We have made this edit.**

2. Please structure your abstract using the PLOS Medicine headings (Background, Methods and Findings, Conclusions).

**Author Response: Thank you. We have made this edit.**

3. Abstract Background: The final sentence should clearly state the study question.

**Author Response: Thank you. We have made this edit.**

4. Abstract Methods and Findings:

- Please ensure that all numbers presented in the abstract are present and identical to numbers presented in the main manuscript text.

**Author Response: Thank you. We have certified that the numbers are present and identical.**

- Please include the important dependent variables that are adjusted for in the analyses.

**Author Response: Thank you. Due to the word limits in the structured abstract, we are not able to include all of the variables adjusted for, but we did add language about the conceptual model that informed our covariate selections.**

- In the last sentence of the Abstract Methods and Findings section, please describe the main limitation(s) of the study's methodology.

**Author Response: Thank you. We have added the following sentence at the end of the methods in the abstract:**

**“Study design limitations include limited PrEP access outside of study, limited follow up during pregnancy, and lack of a control group.”**

5. Please confirm whether author KEW’s affiliation with NoviSci poses a potential competing interest that ought to be declared.

**Author Response: Thank you. We have added the following disclosure:**

**“KEW is an employee and a shareholder of NoviSci/Target RWE, which has received fees from Janssen Research & Development (Janssen R&D) and Amgen Inc. outside the submitted work.”**

6. The author contributions section can be removed (this will be captured as metadata via the Submission Form), and the Acknowledgements section can be relocated to the end of the manuscript.

**Author Response: Thank you. We have made this edit.**

7. Please include continuous line numbering throughout the manuscript.

**Author Response: Thank you. We have made this edit.**

8. At this stage, we ask that you include a short, non-technical Author Summary of your research to make findings accessible to a wide audience that includes both scientists and non-scientists. The Author Summary should immediately follow the Abstract in your revised manuscript. This text is subject to editorial change and should be distinct from the scientific abstract. Please see our author guidelines for more information: https://urldefense.com/v3/__https://journals.plos.org/plosmedicine/s/revising-your-manuscript*loc-author-summary__;Iw!!NoSwA-eRAg!D6jYucxFcctkZhD0lRWfO2ofe_AwJUa061ecS529FfIIwbLvDgwxAqDLgVSbsZZlU87-VNuDl5nx08S8tMnM7g$

**Author Response: Thank you, we have included this after the abstract.**

9. Please place citations in square brackets throughout.

**Author Response: Thank you. We have made this edit.**

10. Please ensure that the study is reported according to the STROBE guideline, and include the completed STROBE checklist as Supporting Information. Please add the following statement, or similar, to the Methods: "This study is reported as per the Strengthening the Reporting of Observational Studies in Epidemiology (STROBE) guideline (S1 Checklist)."

The STROBE guideline can be found here: https://urldefense.com/v3/__http://www.equator-network.org/reporting-guidelines/strobe/__;!!NoSwA-eRAg!D6jYucxFcctkZhD0lRWfO2ofe_AwJUa061ecS529FfIIwbLvDgwxAqDLgVSbsZZlU87-VNuDl5nx08Qgq9hGbQ$

When completing the checklist, please use section and paragraph numbers, rather than page numbers.

**Author Response: Thank you. We have completed the checklist.**

11. Did your study have a prospective protocol or analysis plan? Please state this (either way) early in the Methods section.
    a) If a prospective analysis plan (from your funding proposal, IRB or other ethics committee submission, study protocol, or other planning document written before analyzing the data) was used in designing the study, please include the relevant prospectively written document with your revised manuscript as a Supporting Information file to be published alongside your study, and cite it in the Methods section. A legend for this file should be included at the end of your manuscript.
    b) If no such document exists, please make sure that the Methods section transparently describes when analyses were planned, and when/why any data-driven changes to analyses took place.
    c) In either case, changes in the analysis-- including those made in response to peer review comments-- should be identified as such in the Methods section of the paper, with rationale.

**Author Response: Thank you. We have defined this in the Methods section “Study Procedures,” as shown below:**

**“The protocol and analysis procedures were defined prior to participant enrollment [Periconception PrEP for HIV-exposed Ugandan Women: ClinicalTrials.gov Identifier: NCT03832530].” We have added language to the methods where our analysis diverges from the original protocol.**

12. Please cite specific items located in the Supporting Information as outlined here (as opposed to stating ‘Appendix’ only): https://urldefense.com/v3/__https://journals.plos.org/plosmedicine/s/supporting-information__;!!NoSwA-eRAg!D6jYucxFcctkZhD0lRWfO2ofe_AwJUa061ecS529FfIIwbLvDgwxAqDLgVSbsZZlU87-VNuDl5nx08RirrOiZQ$

**Author Response: Thank you. We have added citations for the Supporting Information documents throughout according to the guidelines at the URL above.**

13. Please specify whether informed consent was written or oral.

**Author Response: Thank you. We have specified that informed consent was written:**

**“All enrolled women provided written informed consent and felt able to attend study visits for the duration of the study.”**

14. Please place each table in your manuscript file directly after the paragraph in which it is first cited (read order). Do not submit your tables in separate files.

**Author Response: Thank you. We have made this edit.**

15. ‘Periconception PrEP Adherence’ and ‘Periconception Drug Concentrations’: PLOS does not permit data not shown – please include the relevant data (in the Supporting Information if necessary).

**Author Response: Thank you. These data are now available in S3 – Supporting Information 3.**

16. Some references are lacking information (e.g., 12-15 and 101-102 should contain URLs and dates of citation where appropriate). See https://urldefense.com/v3/__https://journals.plos.org/plosmedicine/s/submission-guidelines*loc-references__;Iw!!NoSwA-eRAg!D6jYucxFcctkZhD0lRWfO2ofe_AwJUa061ecS529FfIIwbLvDgwxAqDLgVSbsZZlU87-VNuDl5nx08QA4rfr-Q$ for more information.

**Author Response: Thank you. We have updated our citations.**

17. Reference 32 is a preprint – please confirm it has not yet been published and, if not, please add [preprint] to the reference after ‘medRxiv’.

**Author Response: Thank you. We have updated our citations to include the recently published manuscript instead of the preprint.**

**Comments from the Reviewers:**

Reviewer #1

This paper reports an observational study of an intervention to encourage PrEP initiation amongst women planning pregnancy in Uganda. The paper shows high adherence to the intervention. The statistical methods used are appropriate, although some extra detail is recommended (see below). I have some comments, which I have listed below.

1. Title: should mention the study design (i.e. prospective cohort study)

**Author Response: Thank you. We have made this edit.**

2. Abstract: it's not too clear from the abstract what the main study objectives were and how these link to the results: referring to the healthy families intervention but not to the effect of the intervention made the abstract a bit confusing (in isolation) to me.

**Author Response: Thank you. We have made this edit.**

3. Methods: as per above comment it would be good to have a (small) subsection that describes the main objectives of the study.

**Author Response: Thank you. We have made this edit.**

4. Methods: It would be good to have some text that describes how the sample size was chosen. This may not have been chosen on statistical grounds, perhaps on feasibility grounds.

**Author Response: Thank you. We have added this in a new section under Methods:**

**“Power and sample size**

**We proposed to enroll 150 women to have an evaluable sample of 112 women initiating PrEP. Our estimated evaluable sample size was based on preliminary data suggesting that 75% of women will choose to initiate PrEP. However, challenges with recruitment related to national stock-outs of HIV testing supplies resulted in halting enrollment prior to reaching this sample size. However, due to higher than anticipated PrEP use we had sufficient sample size to evaluate factors associated with PrEP adherence.”**

5. Methods, statistical analysis: it would be useful to add whether the statistical analysis was pre-specified and, if so, provide a statistical analysis plan.

**Author Response: see response to editor comment #11 above**

6. Methods, statistical analysis: I found the model used for secondary adherence analysis hard to visualise - could more be added in the appendix on this and how to interpret the parameters?

**Author Response: Thank you, we have added additional text. As described in the Methods section, we estimated mean (95% CI) monthly adherence to PrEP using a stratified, intercept-only modified Poisson regression with generalized estimating equations.**

**To evaluate potential predictors of PrEP adherence, we applied the same multivariable model-building approach used for the primary endpoint. That is, we started with a fully adjusted model which included the covariate of interest and all potential confounders (identified based on subject matter knowledge of the underlying causal structure). We then removed each potential confounding factor one by one and recorded the estimated log relative risk (RR) for the predictor-outcome association of interest. If the removal of the factor changed the log RR by ≥10%, it was retained in the final multivariable model. This model-building process was carried out for each predictor of interest. Table 2 presents the final RR for each predictor of interest whereas S2 – Supporting Information 2 presents the full model results (i.e., predictor plus all retained confounding factors). We do not recommend causally interpreting the regression coefficients for the retained confounding factors as each model was constructed based on the predictor of interest and not any other factor.**

7. Results: I believe PLOS medicine does not permit 'data not shown' so these results could be provided in supplementary material. I think the adherence data are useful to provide as relevant to other research studies.

**Author Response: Thank you. All data are now presented in the text, tables, or supporting information.**

8. Discussion: it would be useful to discuss how representative the participants who enrolled in the study were of the broader target population. Are results from this study likely to be relevant in the broader population?

**Author Response: Thank you for this observation. Our response to this is found in response to the Academic Editor queries in an earlier section of this document.**

Reviewer #2

Thank you for allowing me to look at this manuscript. It has very important results useful to low resource-settings with high HIV prevalence and high fertility settings.

1. Page 12: The ranges overlap. If the participant of 4 doses:

TFV-DP reflects average dosing in the last 6-8 weeks; >600 fmol/punch indicates approximately >4 doses per week (categorized as high adherence), 450-600 fmol/punch indicates about 3- 4 doses per week (categorized as moderate adherence), and bet

**Author Response: Thank you we have corrected this error in the description of the translation from categories of drug concentrations to estimated doses taken per week:**

**“Plasma TFV concentrations >40ng/mL indicate dosing in the last 24 hours (categorized as “high” adherence), between 10 and 40 ng/mL indicates dosing in the last 3 days (categorized as “moderate” adherence), and between >0.31 and 10 ng/mL indicates dosing in the last week (categorized as “low” adherence) [20, 63]. TFV concentrations below 0.31 ng/mL are below the limits of detection. In contrast to data from men [64-67], data on how to assign categories of TFV-DP concentrations to pill taking behavior for women who are not pregnant nor post-partum women in African settings is evolving [66, 68]. Based on the available data and the expertise on this authorship team, >600 fmol/punch was categorized as indicating approximately >4 doses per week (categorized as high adherence), 450-600 fmol/punch indicates about 3 doses per week (categorized as moderate adherence), and between 31.3 and <450 indicates <3 doses per week (categorized as low adherence) [60, 67, 69, 70]. TFV-DP concentrations below < 31.3 fmol/punch are below limits of detection.”**

2. Pregnancy incidence: Please explain how you get the pregnancy incidence of 75% at (53 pregnancies of 131 women)

A total of 53 pregnancies occurred among 131 women with 848 person-months of follow-up (median 8.8 months), resulting in a pregnancy incidence of 75% (95% CI: 57%, 98%) (Figure 2).

**Author Response: Pregnancy incidence was calculated as the number of pregnancies per person-year of follow-up or 53 / ( 848 / 12) = 0.75. We agree that presenting incidence rates as percentages can be confusing. We have modified the text to clarify the incidence calculation, including presenting the 1-year cumulative incidence implied by the incidence rate.

“Among 131 enrolled women, 17 (13%) moved or were otherwise lost to follow-up. A total of 53 pregnancies occurred among 131 women with 848 person-months of follow-up (median 8.8 months), resulting in an incidence of 75 pregnancies per 100 person-years (95% CI: 57, 98) for a 1-year cumulative incidence of 53% (95% CI: 43%, 62%) (Figure 2).”**

3. Page 15: Periconception Drug Concentrations.

All processed samples were from women who were not pregnant at the time of collection.

Please explain why you used only not pregnant participants for this sample collection. Could it be different than that from those who got pregnant?

**Author Response: Plasma and whole blood TFV and TFV-dp levels drop precipitously in pregnancy in part due to increases in plasma volume and cardiac output, thus, rendering existing adherence benchmarks no longer valid; the significance of this for prevention is unclear. It is hard to add these data to this paper easily and carefully within the word limits and therefore have not done so. We have a very small number of samples from pregnant women and plan to aggregate that data with data from another study to more deeply explore drug levels in pregnancy with ample room to discuss our findings.**

4. I expected some explanations of this in the discussion:

One participant tested positive for HIV at 9 months. This participant was not pregnant. Although electronic adherence data suggested high PrEP adherence (>80% of doses all months), plasma TFV levels were undetectable at 3, 6, and 9 months of follow-up.

**Author Response: Please see the response to R3, point 2, below.**

5. Typos. Need to read through the document and correct the typographic errors

e.g ….. counselors (social desirability bias)(85). However, studies have shown that adhering to pill cap opening without taking medication is difficult to maintain over time(86); further, PrEP use was not required to remain in the study.

**Author Response: Thank you. We have re-read the manuscript to address any outstanding typos.**

Reviewer #3

1. I commend the authors for this manuscript that evaluates an are of great public health importance. The study explores important application of HIV prevention intervention in reproductive health programs. The manuscript is well written and has used vigorous analysis approaches to evaluate uptake and adherence of PrEP in the periconception period. The numbers are relatively small and thus denies the investigators ability to draw some conclusions in some scenarios where they acknowledge being underpowered. This may be the major shortcoming of this study, however this is not fatal to their work.

**Author Response: Thank you.**

2. I was surprised with the findings on the participant who seroconverted in the course of the study. This participant had high adherence by electronic adherence data however, her plasma levels were undetectable at month 3, 6 and 9. This, coupled with the results showing poor correlation between TFV-DP and electronic adherence data suggests caution in interpretation of results involving electronic adherence data. I think this may be a contribution of this work in understanding adherence. Would the authors consider firming up their position on this in their manuscript? They appear to remain non-committal.

**Author Response: Adherence inherently involves a degree of uncertainty, and reliance or confidence in a single measure can lead to misinterpretation. The apparent inaccuracy of electronic pillbox adherence with the one participant who acquired HIV does not invalidate the measure, which can be useful in understanding patterns of adherence (e.g., Haberer, JAIDS, 2021; Musinguzi, AIDS Behav 2022). We have expanded the discussion on this point and again highlighted that a strength of the manuscript is using several different measures of adherence.**

**“Differences observed across the PrEP adherence measures in this cohort highlight the value of incorporating multiple adherence measurements and expose some gaps in our understandings of tenofovir drug-level data interpretation for cis-gender women. Importantly, adherence by any one of these measures is higher than seen in many prior cohorts of women of reproductive age. Further, one HIV seroconversion was observed despite high pregnancy and STI incidence indicating that the intervention supported women to avoid HIV transmission over time [85]. Exploring possible reasons for discrepancies across measures is important for interpreting the effect of our intervention and informing future analyses. First, the categories of high are not equivalent across the measures in terms of dosing or time period: 80% of pills taken by electronic pillcap equates to 5-6 doses per week averaged over ~90 days, >40ng/mL represents dosing in the last 24 hours, and >600 fmol/punch TFV-DP represents 4 or more doses per week over 6-8 weeks. In addition, the samples represent different groups: pillcap data were available for 83%, plasma data were processed for 39%, and whole blood samples were processed for 93% of women accessing PrEP at the 3-month (primary outcome) timepoint. High adherence by electronic device persisted whereas the proportion with high levels waned over time with plasma and whole blood measures. Women may have persisted using the pillcap device without taking pills to please the researchers or counselors (social desirability bias)[86]. However, studies have shown that adhering to pillcap opening without taking medication is difficult to maintain over time[87]; further, PrEP use was not required to remain in the study. Plasma TFV measures pill-taking in the past week and women may have been prompted to take doses by reminders about upcoming clinic visits thus explaining higher plasma levels even when TFV-DP concentrations were lower; however, correlations between plasma TFV and electronic pillcap data suggest that pillcap use over 30 days aligned with an objective, biologic measure in the week prior to blood draw. Correlations between electronic pillcap and whole blood (DBS) were low. In a secondary analysis of whole blood spots collected from men and women with electronic pillcap data in the Partners PrEP demonstration project, TFV-DP measurements were specific but not sensitive for electronic pillcap adherence [68] with sensitivity lowest for the highest categories of MEMS adherence. Those authors speculate that the discrepancy may relate to interindividual differences in drug disposition [88], [89, 90]. While inter-individual differences in PK are certain, intra-individual PK changes over time are less likely (particularly with respect to anemia, BMI and other biological factors impacting drug disposition.) Thus, the downward trends of DBS TFV-DP and plasma TFV values likely at least partially reflect behavioral fatigue with daily pill taking and highlight the importance of longitudinal adherence support for daily PrEP use. The most conservative estimates of adherence also suggest that some women may require different adherence support than what was provided in this study and ongoing work is needed to optimize adherence support for women [73, 91]. Again, adherence by all measures was higher in this cohort than for most PrEP implementation cohorts among women: the most conservative adherence benchmark is the TFV-dp concentration and 47%, 41%, and 45% of women at months 3, 6, and 9 attained these high levels in this compartment.”**

3. Finally, in the discussion, on a paragraph that appears with ref #45 and #79 rectify this typo error “to predict which women” and NOT “to predict who which women.”

**Author Response:** **Thank you, this has been corrected.**

Reviewer #4

This is an interesting manuscript about an important priority population for HIV prevention - women with periconception risk of HIV acquisition. There is discussion of an exciting new intervention as well as very robust measurement of adherence to PrEP using multiple measures and the authors should be congratulated on the work. Overall, the adherence to PrEP in this study was impressively high compared to other recent cohorts and deserves attention by the field. However, I do think that the overall focus of the manuscript could be clarified and strengthened to make this paper more accessible to readers. I hope the comments below are helpful to the authors in revising the manuscript.

**Author Response: Thank you, we appreciate the comment and hope the revised manuscript is clearer that we are focusing on the adherence measurements in this cohort. We have published on the acceptability and feasibility via IDIs for the counseling components of the Healthy Families intervention and the adherence counseling is based in a CDC EBI.**

Major comments:

1. In terms of the overarching focus of the manuscript it was somewhat unclear to me whether the focus was on the development and evaluation of impact of a novel intervention or on a detailed comparison of adherence metrics within the cohort. The abstract (Methods) states women were enrolled to evaluate impact of intervention but there is no comparator group against which to evaluate. Each of these questions (intervention and adherence metrics) is interesting and it would have been helpful to more clearly describe/separate in abstracts/results/discussion. I was also interested in more data on how the intervention was delivered/by whom, how long it took, acceptability of sessions - if this is to be evaluated further and potentially scaled up. If the focus is on the intervention, would suggest including figure of conceptual model in main paper if possible or in supplemental figures, and also including more on the intervention in the Discussion

**Author Response: Thank you. We now provide more details in the manuscript regarding the details of the intervention and reference prior publications that assess acceptability and feasibility from the perspective of clients and providers. We also clarify that the adherence support component of the intervention is an evidence-based intervention which we adapted to this setting. Due to the modest funding support and nature of prior data, this small cohort did not include a lot of process indicators (e.g. fidelity check lists, recordings of sessions and feedback, feasibility/acceptability questionnaires), but we plan to integrate these process metrics and implementation outcomes into a future hybrid effectiveness/implementation hybrid evaluation for the intervention. We have added the gaps and future plans to the discussion.**

***Introduction - final paragraph:***

**Based on formative work and informed by a conceptual framework for periconception HIV-exposure behavior [35], we developed a counseling support intervention, Healthy Families-PrEP, for HIV-negative women of reproductive age in Uganda with personal or partner plans for a pregnancy in the next year. Healthy Families-PrEP leverages individual- and couple-level reproductive goals to promote uptake and use of HIV prevention strategies, including TDF/FTC PrEP. In prior publications we described high client- and provider-level acceptability of the counseling program when integrated into routine HIV care {Young, 2021 #36}. Here, we show how women participating in this combination counseling intervention used PrEP during periconception and pregnancy periods.**

***Methods: Study Procedures – paragraph 2:***

**The Healthy Families-PrEP intervention was informed by a socio-ecological framework to understand women’s PrEP use [44, 45], and our periconception risk behavior conceptual framework [35]. Healthy Families-PrEP leveraged individual- and couple-level reproductive goals to promote uptake and use of HIV prevention strategies through education, problem-solving, communication-skills training, and adherence support. Counselors completed a two-day in-person training (together with a research team in South Africa {Matthews, 2019 #105} and quarterly supervision calls with members of the study team (CP and post-doctoral fellows). Trained counselors used an intervention guide and were directed to complete a 60-minute introductory session followed by 30-minute follow-up sessions in which they worked with participants to develop and implement a safer conception plan. For women who did not know their partner’s HIV status, this plan included how to encourage their partners to test and disclose their status. For those with partners living with HIV, counselling included how to encourage partners to initiate ART, to delay condomless sex until partner achieved either ART coverage for 6 months or HIV viral load suppression, and to limit sex without condoms to peak fertility. Oral combination TDF (300mg) /FTC (200mg) as Truvada was offered as PrEP and additional adherence counseling support was included at quarterly visits as long as the participant chose to use PrEP based on methods developed by Dr. Psaros and colleagues and included in the CDC compendium of evidence based interventions [47, 48].**

***Discussion – paragraph 2:***

**The Healthy Families PrEP combination intervention offered counselling delivered by local lay counselors to support HIV prevention strategies including daily PrEP use in the context of planning for or being pregnant (Figure 1). The three counseling sessions offered to all women were developed to be offered to individuals or couples and acceptability and feasibility from the standpoint of clients and providers is published {Young, 2021 #36}. Quarterly adherence support was adapted from the Lifesteps intervention, an evidence-based intervention included in the CDC compendium of effective interventions for HIV prevention, and utilized education, problem solving and motivational interviewing strategies to promote adherence. Lifesteps was first adapted for PrEP among HIV-serodifferent couples in Uganda and successfully promoted PrEP adherence [46]. Here the same tenets were applied with an added emphasis on the opportunity to prevent HIV acquisition while meeting reproductive goals. We did not collect detailed process data, but counselors were trained to offer sessions lasting 30-60 minutes. Our team provided in-person training and every other month supervision support, where counselors discussed challenges. While this counseling approach was effective for most women in our cohort, some encountered ongoing adherence challenges. Future iterations of this work should aim to predict which women are most likely to experience adherence challenges, and identify what role adherence counseling may play in supporting prevention effective PrEP use, regardless of PrEP formulation (e.g. adaptive interventions [74]). For women who consistently use PrEP, adherence counseling support could be scaled down. We are applying for funding to support a full effectiveness trial and preliminary exploration of implementation strategies in collaboration with the Ugandan Ministry of Health in order to inform best approaches for public sector implementation and future scaling to meet the needs women of reproductive age. In these next steps, we will evaluate the effectiveness and integration of this approach into routine care to improve PrEP uptake and adherence, including the cost, feasibility, scale-ability and implementation climate/process in Uganda.**

2. Regarding the adherence data, the adherence via objective metrics was quite high in this study compared to other studies in similar populations/settings. However, given that multiple studies have suggested that electronic pillbox openings overestimate adherence and (as authors note) plasma reflects only very recent adherence - would suggest that the authors focus on the TFV-DP metrics as a cumulative metric more reflective of adherence over time in the study (recognizing that not all participants had DBS measures). Even using the TFV-DP metric, about 45% with high adherence and 60% with any adherence at month 9 is still much higher than in many recent studies - although this was not a primary outcome would suggest emphasizing levels of adherence in study around these #s rather than 80% which is likely an overestimate. It is good that the authors acknowledge some uncertainty around appropriate cutoffs for TFV-DP in DBS for African women and would suggest bringing this point in earlier when the thresholds are described in the methods.

**Author Response: We appreciate the reviewer’s comments. All adherence measures have strengths and limitations. We have seen overestimation with the electronic pill box compared to TFV-DP in DBS; however, underestimation is also possible (e.g., taking the medication from another container or taking multiple pills out at once for later dosing). Interpretation of pharmacokinetic levels (i.e., plasma and dried blood spots) can also be challenging, particularly in pregnancy (e.g., Pyra, AIDS 2018 Aug 24;32(13):1891-1898). Please see Musinguzi *et al*. (AIDS. 2016 Apr 24;30(7):1121-9 and AIDS 2018, 32:2237–2245) for detailed comparisons of adherence measures regarding PrEP use.**

**Given the potential for uncertainty in adherence measurement, we think the current focus on multiple approaches is a strength of the paper and respectfully retain all three measures. We have added language throughout to emphasize the value in the different measures and have added summary data on the TFV-dp findings to the abstract. We now highlight the reviewer’s concerns and our perspective earlier in the introduction, methods, and discussion.**

**Text related to this comment is included below by section:**

***Abstract - Conclusions:***

**Women in Uganda with PrEP indications and planning for pregnancy chose to use PrEP. By electronic pillcap, most were able to sustain high adherence to daily oral PrEP prior to and during pregnancy. Differences in adherence measures highlight challenges with adherence assessment and serial measures of TFV-dp in whole blood suggest 41-47% of women took sufficient periconception PrEP over weeks to prevent HIV. These data suggest that women planning for and with pregnancy should be prioritized for PrEP implementation, particularly in settings with high fertility rates and generalized HIV epidemics.**

***Methods: Assessment of PrEP Uptake and Adherence – paragraphs 2 & 3:***

**We used multiple approaches to estimate PrEP adherence given that all adherence measures have advantages and limitations. Specifically, electronic monitoring provides highly informative day-to-day patterns of adherence; however, it may under or overestimate adherence due to device non-use or curiosity openings of the pillbox without dosing, respectively. Biomedical assessments of PrEP adherence provide documentation of pill ingestion, yet they can only be collected at periodic time points due to resource and logistical constraints, and interpretation of drug concentrations may be complicated by variable metabolism, drug interactions, and/or laboratory variation. Notably, the time period reflected by each matrix is variable (i.e., ~7 days for plasma, ~8 weeks for erythrocytes after achievement of steady state). We therefore determined plasma TFV and dried blood spot (DBS) intraerythrocytic TFV-DP concentrations for a subset of participants.**

**Plasma TFV concentrations >40ng/mL indicate dosing in the last 24 hours (categorized as “high” adherence), between 10 and 40 ng/mL indicates dosing in the last 3 days (categorized as “moderate” adherence), and between >0.31 and 10 ng/mL indicates dosing in the last week (categorized as “low” adherence) [20, 65]. TFV concentrations below 0.31 ng/mL are below the limits of detection. In contrast to data from men [66-69], data on how to assign categories of TFV-DP concentrations to pill taking behavior for women who are not pregnant nor post-partum women in African settings is evolving [68, 70]. Based on the available data and the expertise on this authorship team, >600 fmol/punch was categorized as indicating approximately ≥4 doses per week (categorized as high adherence), 450-600 fmol/punch indicates about 3 doses per week (categorized as moderate adherence), and between 31.3 and <450 indicates <3 doses per week (categorized as low adherence) [58, 69, 71, 72]. TFV-DP concentrations below < 31.3 fmol/punch are below limits of detection.**

***Discussion – paragraph 7:***

**Again, adherence by all measures was higher in this cohort than for most PrEP implementation cohorts among women: the most conservative adherence benchmark is the TFV-dp concentration and 47%, 41%, and 45% of women at months 3, 6, and 9 attained these high levels in this compartment.**

Additional comments:

*Abstract*

4. Could the authors please briefly describe setting from which participants were recruited and where visits occurred (e.g. general ANC clinic/hospital/community)?

**Author Response: Thank you. Unfortunately, do to word count restrictions according to journal policies, we were unable to describe the settings from which participants were recruited. The abstract reads:**

**“We enrolled HIV-negative women with plans for pregnancy with a partner living, or thought to be living, with HIV (2017-2020) to evaluate PrEP use among women participating in the Healthy-Families intervention.” We explain the recruitment in more depth in methods.**

*Methods*

5. Methods - consider rewording "impact" of intervention given no comparator (pre/post or control arm)

**Author Response: Thank you. We have removed the word “impact” to make the statement clearer. It now reads:**

**“…to evaluate PrEP use among women participating in the Healthy Families intervention.”**

6. Population and eligibility (top of page 9) - how was self-assessed risk determined

**Author Response: Thank you. We have clarified by amending the sentence to say the following:**

**“Additionally, an eligible woman either knew her pregnancy partner was living with HIV or felt she was at risk for acquiring HIV based on the Perceived Risk of HIV Scale [42].”**

7. Healthy living intervention Figure 1- text at bottom of figure states 12 weeks from enrollment to completion of all sessions but box on Sessions 2,3 states delivered every 3 months - could the authors clarify how often sessions were delivered and total follow-up time to deliver the intervention?

**Author Response: Thank you. We have clarified the timing in the intervention figure and in the methods.**

8. Study procedures - participants received a package of HIV prevention OR safer conception counseling - were both offered or only one (should this be AND?)

**Author Response: Thank you we have clarified that the HIV prevention package is a safer conception package.**

9. Middle of page 10 - baseline HIV testing - could the authors kindly confirm if this was POC 4th gen Ag/Ab or rapid 3rd gen Ab? To my knowledge 3rd gen is usually SOC in this setting rather than POC 4th gen but would appreciate clarification.

**Author Response:** **Thank you for catching this. The screening algorithms used rely on antibody testing only, not 4th generation testing. We have corrected this error in the manuscript.**

10. Middle of page 12 - discussion of thresholds for TFV-DP - would be helpful to mention here that the 600 fmol/punch differs from prior thresholds in earlier studies e.g. MSM and how 600 was selected

**Author Response: Thank you. We have added a bit about the basis for our selections to the methods. We offer a more detailed explanation in the discussion:**

**“****Whole blood concentrations of TFV-DP associated with protection from HIV for women are not available. Twenty women from East and Southern Africa who were 6-12 weeks post-partum and taking directly observed oral PrEP 7 times per week had 25^th^ percentile concentrations of TFV-DP of 1053 fmol/punch [71], and the corresponding estimate for 4 doses per week was 600fmol/punch. For 25 U.S. women (5 of whom were Black, none noted to be pregnant), the 25^th^ percentile concentration for steady state dosing at 4 doses per week was approximately 700 fmol/punch [70]; these data, plus data from men, has informed use of a concentration of 700 fmol/punch to indicate high adherence [68-71]. However, because data suggest that this cut-off is not sensitive for adherence in African cis-gender women [72], we used 600 fmol/punch.”**

11. Statistical analysis - for the adjusted model - was adjustment based on the initial causal model or stepwise removal?

**Author Response: The fully adjusted model was initially constructed based on subject-matter knowledge regarding known and/or hypothesized confounding factors (i.e., causal structure). After specifying the fully adjusted model, we used a stepwise removal process based on the log RR to remove those factors that did not impact the effect estimate of interest. That is, given a finite sample size, we sought to maximize the statistical efficiency of the model while preserving our ability to control for the measured confounding factors identified by the causal structure. We note that the stepwise removal procedure we used did not rely on p-values as this approach is problematic for control of confounders.**

*Results*

12. Periconception drug concentrations - were any blood samples collected from women who were pregnant? Is figure 4 only showing data from women who were not pregnant, e.g. at 9 months of follow-up? I understand initially women were exited from study at time pregnancy identified but adherence during pregnancy would also be of interest and expected to see some data here - thank you for insights/clarification

**Author Response: Because pregnancy PrEP follow-up was only added to the protocol partway through the study, we have a small number of samples from pregnant women accessing PrEP. Plasma and whole blood TFV and TFV-dp levels drop precipitously in pregnancy in part due to increases in plasma volume and cardiac output, thus, rendering existing adherence benchmarks no longer valid; the significance of this for prevention is unclear. In order to properly explore the drug concentration data (paired with pillcap data) we plan to combine our Uganda cohort data with similar data from another study of periconception and pregnancy PrEP use to more deeply explore drug levels in pregnancy. We felt including these data with a proper exploration of the findings and interpretation was beyond the scope of this paper. We have mentioned this in the discussion:**

**“We have limited tenofovir concentration data from pregnant people in this study and will be conducting a future combined analysis with another dataset.”**

13. Middle of page 15 - on drug levels - authors state comparison of electronic and plasma data .. "suggesting that pill taking behavior over longer time frames correlates well with objective adherence behavior reflecting pill taking over the past week" - would consider revising this sentence given the subsequent DBS data and that electronic pillbox openings does not necessarily mean pill ingestion. As noted later in paragraph, electronic data did not correlate with TFV-DP levels.

**Author Response:** **Thank you. We have edited this phrasing to “Correlations at 6 and 9 months were also moderate to high and statistically significant (data not shown), suggesting that pill-taking behavior correlates well with objective adherence behavior over the past week.”**

14. Figure 3b seems to come after Figure 4 - possible to renumber for clarity?

**Author Response: Thank you. We now present this as Figure 5.**

*Discussion*

15. Consider emphasizing (as in half-way through paragraph 1) that about half of women consistently took oral PrEP based on TFV-DP - agree that this is still quite a high percentage

**Author Response: Thank you. We have included additional text regarding the significance of the TFV-dp concentrations as well as the plasma and pillcap data in this cohort.**

16. In Paragraphs 2 and 3 would have been interested in more emphasis on the results of this study rather than results from other studies

**Author Response: Thank you. We have updated the discussion as recommended.**

17. Paragraph 4 on intervention - would have been interested (as noted above) in more results and discussion of this novel intervention

**Author Response: Thank you. We have updated the discussion as recommended.**

18. Conclusion - I agree with all of the statements in general, e.g. women planning for pregnancy should be prioritized for PrEP - but would appreciate more clarity on how the results of this study lead to the conclusion.

**Author Response: Thank you, we have updated the conclusion as recommended.**
