# Supplementary material for: High PrEP uptake and objective longitudinal adherence among HIV-exposed women with personal or partner plans for pregnancy in rural Uganda: A cohort study
Source: PLoS Med. 2023 Feb 16;20(2):e1004088. doi: 10.1371/journal.pmed.1004088 (PMC9983833; doi:10.1371/journal.pmed.1004088)
Supplement: Attachment — Submitted filename: RTR 17Jan2023 final.docx [file pmed.1004088.s008.docx]

Response to Review

17 January 2023

1. Please review your reference list to ensure that it is complete and correct. If you have cited papers that have been retracted, please include the rationale for doing so in the manuscript text, or remove these references and replace them with relevant current references. Any changes to the reference list should be mentioned in the rebuttal letter that accompanies your revised manuscript.

**Thank you. We updated formatting for all of the references, included dates of access for webpages, and updated the published manuscripts of those that were previously marked “In press.”**

2. You note in the Acknowledgements that Gilead Sciences reviewed the final manuscript – please include this information in your Financial Disclosure and Competing Interests. Additionally, please indicate whether Gilead Sciences played any other role in the study design, data collection and analysis, decision to publish, or preparation of the manuscript?

**Thank you, we have made this edit. Disclosures now say:**

*Gilead Sciences provided study drug and reviewed the final manuscript, but had no role in the study design, data collection and analysis, decision to publish, or preparation of the manuscript.*

3. Please structure your Abstract using PLOS Medicine headings: Introduction, Methods and Findings, Conclusions (the Methods and Findings sections ought to be combined).

**Thank you, we have made this edit.**

4. Please relocate the sentence ‘Study design limitations include lack of a control group’ to the end of the Methods and Findings section (final sentence before Conclusions section).

**Thank you, we have made this edit.**

5. Please include continuous line numbering throughout the manuscript (in the last revision this only appeared in the .doc version and not the PDF).

**Thank you, we have made this edit.**

6. The Disclosures and Funding sections can be removed from the main text, please ensure all details are captured in the relevant answer from the submission form (Competing Interests).

**Thank you, we have ensured they are captured in the submission form.**

7. Please format your Author Summary using 2-3 single sentence bullet points per question.

**Thank you, we have made this edit.**

8. Please provide legends for all Figures (including those in the Supporting Information).

**Thank you. We have made sure to include legends for all figures.**

**We have provided the individual figure files as .tif format files, and we have included .docx files with each figure including its title and legend. Please let us know if more detail is needed.**

9. Please define ‘lost to follow up’ as used in this study.

**Thank you, we have made this edit and included the definition in the results.**

10. Table 2: Please include the covariates adjusted for in the legend.

**Thank you. The table has been updated accordingly.**

11. Please enlarge the text in Figure 4 as it is currently difficult to read.

**Thank you, we have made this edit.**

12. In the interest of brevity and clarity, please trim the additional Discussion text relating to transmission despite high adherence.

**Thank you, we have lightly edited some of the text that was added to address specific reviewer concerns about this issue.**

13. References: Please include date cited for any internet sources.

**Thank you, we have made this edit.**

14. Please delete the competing interests information from references 28 and 61.

**Thank you, we have made this edit.**

**Additional Changes:**

**We have lightly edited the biostatistical analysis section of the methods for clarity. Neither the output nor the approach are changed.**

Comments from Reviewers:

1. Reviewer #1: Thank you to the authors for addressing my previous comments well. I have no further issues to raise.

**Thank you.**

2. Reviewer #4: The authors were highly responsive to the reviews. I appreciate the opportunity to re-review the manuscript and the additional details on the intervention are very helpful. I recommend acceptance with if one suggested change as follows in the Abstract – The objective has been changed from "We conducted a longitudinal cohort study in Uganda to evaluate oral PrEP uptake and adherence as part of HIV prevention in the context of reproductive goals for women (i.e., safer conception care)." to "We conducted a longitudinal cohort study to evaluate whether Healthy-Families-PrEP supported sufficient oral PrEP use to reduce HIV incidence among women." The revised sentence including the phrase "to reduce HIV incidence" is somewhat misleading because HIV incidence is not the primary outcome of the study and the sample size is rather small for incidence measurement. Would suggest that the authors reword the sentence to focus on uptake and adherence rather than incidence.

**Thank you, we have made this edit. It now says:**

*We conducted a longitudinal cohort study to evaluate oral PrEP use among women participating in the Healthy-Families-PrEP intervention.*
