# Supplementary material for: High PrEP uptake and objective longitudinal adherence among HIV-exposed women with personal or partner plans for pregnancy in rural Uganda: A cohort study
Source: PLoS Med. 2023 Feb 16;20(2):e1004088. doi: 10.1371/journal.pmed.1004088 (PMC9983833; doi:10.1371/journal.pmed.1004088)
Supplement: Attachment — Submitted filename: Reviewer comments.docx [file pmed.1004088.s006.docx]

**High PrEP uptake and objective longitudinal adherence among HIV-exposed women with personal or partner plans for pregnancy in rural Uganda**

Thank you for allowing me to look at this manuscript. It has very important results useful to low resource-settings with high HIV prevalence and high fertility settings.

Comments:

1. Page 12: The ranges overlap. If the participant of 4 doses:

TFV-DP reflects average dosing in the last 6-8 weeks; >600 fmol/punch indicates ***approximately ≥4 doses per week*** (categorized as high adherence), 450-600 fmol/punch indicates ***about 3- 4 doses*** per week (categorized as moderate adherence), and bet

2. Pregnancy incidence: Please explain how you get the pregnancy incidence of 75% at (53 pregnancies of 131 women)

A total of 53 pregnancies occurred among 131 women with 848 person-months of follow-up (median 8.8 months), resulting in a ***pregnancy incidence of 75%*** (95% CI: 57%, 98%) (Figure 2).

3. Page 15: Periconception Drug Concentrations.

All processed samples were from women who were not pregnant at the time of collection.

*Please explain why you used only not pregnant participants for this sample collection. Could it be different than that from those who got pregnant?*

4. I expected some explanations of this in the discussion:

*One participant tested positive for HIV at 9 months. This participant was not pregnant. Although electronic adherence data suggested high PrEP adherence (>80% of doses all months), plasma TFV levels were undetectable at 3, 6, and 9 months of follow-up.*

5. Typos. Need to read through the document and correct the typographic errors

e.g ….. counselors (social desirability bias***)(85).*** However, studies have shown that adhering to pill cap opening without taking medication is difficult to maintain over ***time(86);*** further, PrEP use was not required to remain in the study.
